# Decrease in beneficial bacteria and increase in harmful bacteria in *Gastrodia* seedlings and their surrounding soil are mainly responsible for degradation of *Gastrodia* asexual propagation

**DOI:** 10.3389/fpls.2024.1334958

**Published:** 2024-02-06

**Authors:** Xi Wang, Yugang Gao, Pu Zang, Ge Zhang, Xinyu Yang, Qun Liu

**Affiliations:** ^1^ College of Chinese Medicinal Materials and Laboratory of Medicinal Plant Cultivation and Breeding, National Administration of Traditional Chinese Medicine, Jilin Agricultural University, Changchun, China; ^2^ Institute of Botany, Jiangsu Province and Chinese Academy of Sciences, Nanjing, China

**Keywords:** *Gastrodia elata Bl. f. glauca S. Chow*, Changbai Mountain, asexual reproduction degradation, microbial diversity, isolation and characterization, antagonistic strains

## Abstract

**Introduction:**

Asexual reproduction of *Gastrodia elata Bl. f. glauca S. chow* (GeB) produces degeneration with increasing number of GeB. Therefore, we analyzed the microorganisms of GeB seedlings and surrounding soil by Illumina Miseq high-throughput sequencing technology.

**Methods:**

In this study, Illumina Miseq high-throughput sequencing technology was applied to analyze the types and quantities of GeB seedlings and surrounding soil microorganisms in the first to third generations of asexual reproduction, isolated and identified the dominant strains of GeB in the first to third generations and screened the antagonistic bacteria of its pathogenic fungi, and evaluated the effects of beneficial bacteria on the production performance of seedlings planted with GeB.

**Results:**

With an increase in the number of asexual reproductive generations, the number of pathogenic fungi and bacteria in GeB seedlings and the surrounding soil increased, and the number of beneficial fungi and bacteria decreased. *Pseudomonas* sp., *Agrobacterium rhizomes*, and *Herbaspirillum hiltneri* were isolated and identified in the first generation, and *Trichoderma harzianum, Penicillium viridiatum, Fusarium oxysporum*, and *Novosphingobium* sp. Were isolated and identified in the third generation. Antagonistic strains of the three pathogenic bacterial strains were screened. In conclusion, beneficial bacteria significantly improved the production performance of asexual reproductive seedlings planted with GeB.

**Discussion:**

In conclusion, our findings suggested that the microorganisms of GeB seedlings and the surrounding soil change as the number of generations of GeB reproduction increases, disrupts the microecological balance of surrounding soil and endophytic microbiomes.This study provides a theoretical basis for the degradation of asexual reproduction in GeB.

## 1 Introduction

The whole family of Orchidaceae has a rich species diversity (more than 28,000 species in the whole family), and the family covers primitive, partial, and complete fungi heterotrophic species (Baasanmunkh et al., 2021). *Gastrodia elata Bl. f. glauca* S. *chow* (GeB) is a non-toxic perennial herbaceous heterotrophic plant with a sweet flavor, smooth texture, and various benefits (Hong et al., 2018; Liu et al., 2018; Gong et al., 2021; Shan et al., 2021; Shan et al., 2021; Bae et al., 2022). GeB has no roots and no green leaves, so it cannot directly absorb water and inorganic salts from the soil for the growth of its plants, nor it can photosynthesize to produce organic nutrients for itself. However, GeB relies on symbiotic fungi to provide the nutrients that it needs for growth, so GeB is a typical fungi heterotrophic parasitic herbaceous plant, which relies on assimilation of some fungi hyphae that invade its body to obtain nutrients. There are at least two kinds of fungi in the life of GeB to provide nutrients for it: one kind of fungi is called *Mycena*, which provides nutrients for the seed when it germinates, so that it grows “mizuna”, and the other kind of fungi is called *Armillariella mellea*, which provides nutrients for GeB.

The demand for GeB has been increasing. However, wild GeB is scarce. Thus, artificial cultivation has been used to fulfill this demand, solving the problem of GeB supply and demand (Wang J. et al., 2023). The GeB life cycle includes the entire growth process of seeds, protocorms, mizuna, seedlings, mature tubers, and seeds (Li et al., 2020b). Seedlings are derived from asexual and sexual reproduction (Su et al., 2023). The sexually reproducing seeds of GeB have strong resistance and high yield but are often limited by seasonality and site (Yu et al., 2023), a long reproductive cycle (Hong et al., 2002), and many production links (Liu et al., 2022). Asexually reproduced GeB seedlings have short growth cycles and fewer seasonal and site-specific limitations (Park et al., 2012) than sexually reproduced GeB seedlings do. However, asexually reproduced seedlings are prone to degradation, resulting in a low yield of GeB (Han et al., 2020). Thus, clarifying the causes and solutions for the degradation of asexually reproductive seedlings is crucial for the production of GeB.

GeB seedlings undergo morphological changes (Park et al., 2012), and yield declines (de Meeûs et al., 2007) after asexual reproduction and passage. However, the underlying mechanism remains unclear. Endophytes in medicinal plants play an important role in host growth and quality (Naranjo et al., 2023) by providing plants with the nutrients that they need (Guan et al., 2022), promoting plant growth (Liu et al., 2020). Rhizosphere fungi and endophytes of the Orchidaceae family provide nutrients to plants (Barnes et al., 2016), enhance plant stress and disease resistance (Huang et al.,2022), and promote seed germination (Zhang et al., 2021; Jiang et al., 2022). Much valuable work has been conducted on other medicinal plant diseases, but studies on GeB seedling degradation from the perspective of microbial community diversity have not been reported.

The aim of this study was to clarify the causes of the degradation of asexual reproduction in GeB seedlings and to find ways to relieve this degradation. GeB seedlings were used as research objects. First, massive amplicon sequencing (Wang Z. et al., 2023; Chen et al., 2019; Clemmensen et al., 2022) was used to analyze the types and quantities of the first to third generations of asexual reproductive GeB seedlings and surrounding soil microorganisms, and the effects of propagation generations on GeB seedlings and their soil microbial diversity were clarified. Second, the isolation and identification of dominant bacteria were conducted, and the microbial community structure was characterized. The types of dominant beneficial fungi and bacteria, pathogenic fungi, and bacteria were clarified, and the antagonistic bacteria of the pathogenic fungi were screened through confrontation experiments. Finally, the effects of beneficial fungi and bacteria on the production of GeB seedlings were evaluated. Thus, this study provides a theoretical basis for exploring the causes and regulation of asexual reproduction and degradation in GeB.

## 2 Material and methods

### 2.1 Sample source

**Figure 1:**
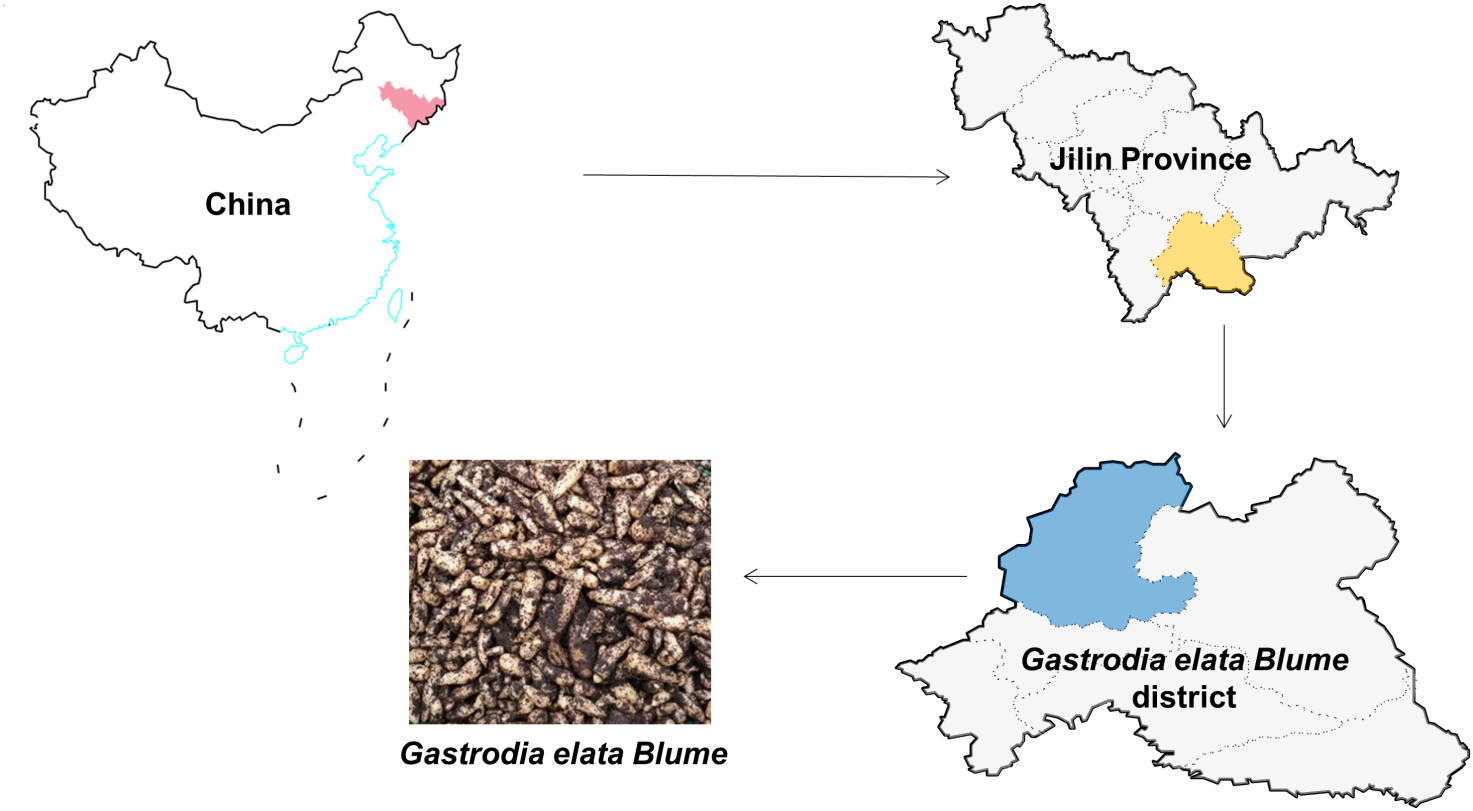
Biogenic information.

GeB seedlings and surrounding soil of different propagation generations of GeB in Changbai Mountain were collected from Dabeishan Village, Longquan Town, Jingyu County, Baishan City, Jilin Province (126°30′E–127°16′E, 42°06′N–42°48′N). The study area has an average elevation of 775 m. It has a monsoon climatic zone and a cold-temperate humid climatic zone in the northeastern mountainous areas. In order to avoid the influence of other factors such as climate on changes in the microbial community, three GeB seedlings with reproductive generations in the same planting area from Baishan Jingzhen Tianma Development Co. (**Figure 1**) were selected for the experiments to ensure that the ground was level. In addition, a thermometer was used for measurement: the temperature was regulated by the density of the shade net, which was maintained at 20°C ± 3°C. In addition, the moisture content of the soil is measured by a hygrometer: the humidity is maintained at 50% ± 5% by artificial watering. This ensures that the study is less exposed to environmental impacts. Five sampling points were selected for the soil, and tubers of the first (first generation of sexual propagation), second (first generation of asexual propagation), and third (second generation of asexual propagation) generations of GeB seedlings by pooled samples were collected using the serpentine sampling method (S sampling method). The S sampling method is as follows: at the determined sampling point, first, use a small soil shovel to remove the surface layer of about 3 cm of soil and, then, tilt down a piece of soil.

### 2.2 Sample processing

Healthy GeB seedlings were selected, and 18 GeB seedlings from different generations were used for microbial community analysis. Soil samples were collected separately from each of the six GeB seedlings and placed in sterile polyethylene bags in the surrounding soil. For every six GeB seedlings, the tubers were cut into small piece; each sample was mixed evenly to prepare a GeB seedling sample. Three replicates were set for each propagation generation, and the seedling and soil samples were replicated.

The soil surrounding the first generation of GeB seedlings was labeled Y, with three replicates (Y1, Y2, and Y3). The soil surrounding the second generation of GeB seedlings was labeled as T, with three replicates (T1, T2, and T3). The soil surrounding the third generation of GeB seedlings was labeled W, with three replicates (W1, W2, and W3).

The first generation of GeB seedlings was labeled A, with three replicates (A1, A2, and A3). The second generation of GeB seedlings was labeled B, with three replicates (B1, B2, and B3). The third generation of GeB seedlings was labeled C, with three replicates (C1, C2, and C3).

### 2.3 Sequence analysis

DNA extraction and massive sequencing of microbiome amplicons from GeB seedlings and their surrounding soil microorganisms were performed by Shanghai Personal Biotechnology Co., Ltd. In brief, the DNA of each sample was extracted using the OMEGA Soil DNA Kit (Omega Bio-Tek, Norcross, GA, USA), using the manufacturer’s instructions. The quantity and quality of the extracted DNAs were measured using a NanoDrop NC2000 spectrophotometer (Thermo Fisher Scientific, Waltham, MA, USA) and agarose gel electrophoresis, respectively. The DNA was stored at 20°C until the PCR step. For bacteria, the part region of the 16S rRNA gene was amplified using the primer pair 338F/806R (V3–V4 region) for the surrounding soils and 799F/1193R (V5–V7 region) for the GeB seedlings (**Table 1**). For fungi, the nuclear ribosomal internal transcribed spacer 1 (ITS1) region was amplified using the primer pair ITS5/ITS2 for the surrounding soils and ITS1F/ITS2 for the GeB seedlings (**Table 1**). Sequencing was performed on an Illumina NovaSeq platform (Illumina, San Diego, CA, USA) to generate 250-bp paired-end reads. All raw data were uploaded to the Sequence Read Archive (SRA), numbers PRJNA1049783, PRJNA1049914, PRJNA1049820, and PRJNA1049958.

**Table 1:** PCR amplicon sequencing.

Primers	Surrounding soil		GeB seedlings	
	ITS1	16S_V3V4	ITS1	16S_V5V7
Forward primers	GGAAGTAAAAGTCGTAACAAGG	ACTCCTACGGGAGGCAGCA	CTTGGTCATTTAGAGGAAGTAA	AACMGGATTAGATACCCKG
Reverse primers	GCTGCGTTCTTCATCGATGC	GGACTACHVGGGTWTCTAAT	GCTGCGTTCTTCATCGATGC	ACGTCATCCCCACCTTCC

### 2.4 Data processing

Microbiome bioinformatics was performed using QIIME2 2019.4, with slight modification, according to the official tutorials (https://docs.qiime2.org/2019.4/tutorials/). In brief, raw sequence data were demultiplexed using the Demux plugin, followed by primer cutting using the Cutadapt plugin. Sequences were then quality-filtered, denoised, and merged, and the chimera was removed using the DADA2 plugin. Non-singleton amplicon sequence variants (ASVs) were aligned with Multiple Alignment using Fast Fourier Transform (MAFFT) and used to construct a phylogenetic tree using fasttree2. Observed species, Shannon, and Simpson indices as well as other indices were calculated. The alpha diversity index was plotted as a sparse curve according to the Chao1 index, and species annotations in the samples were counted using the amplicon sequence operational taxonomic unit (OTU) sequence. Taxonomy was assigned to ASVs by using the classify-sklearn naive Bayes taxonomy classifier in the Silva database (v138). ITS gene selection was performed using the UNITE database (Release 8.0, https://unite.ut.ee/). 16S rRNA gene, select Greengenes database (Rlease 13.8, http://greengenes.2ndgenome.com/). A Venn diagram was generated to visualize the shared and unique ASVs among samples or groups by using R package “VennDiagram,” based on the occurrence of ASVs across samples regardless of their relative abundance (Zaura et al., 2009). Taxa abundances at the ASV level were statistically compared among samples or groups by using MetagenomeSeq and visualized as Manhattan plots (Zgadzaj et al., 2016). The number of taxonomic units contained in each of the seven taxonomic levels of the resultant kingdom, phylum, class, order, family, genus, and species was analyzed by clustering fungi and bacteria and using R software to produce heat maps. Heat map analysis was performed using Genescloud tools, a free online platform for data analysis. A heat map was plotted using heat map tools on the Genescloud platform (https://www.genescloud.cn). The tool was developed using the pheatmap package (V1.0.8), which was slightly modified to improve the layout style. The data were normalized to z-scores. The package uses popular clustering distances and methods implemented in the dist and hclust functions in R. unweighted pair-group method with arithmetic (UPGMA) cluster analysis uses Genescloud tools, a free online platform for data analysis, and Euclidean distances.

### 2.5 Isolation of microorganisms

For the isolation of soil microorganisms around the periphery of GeB seedlings, first to third generations of the peripheral soil of GeB seedlings were passed through a 2-mm–pore size sieve. Next, 1 g of it was weighed and placed in 10 mL of sterilized water. The mixture was allowed to stand for 30 min and then thoroughly mixed. It was diluted 10^3^-, 10^4^-, and 10^5^-fold, respectively; placed in a 10-mL centrifuge tube; and stored in the refrigerator at 4°C for later use.

The isolation of microorganisms of GeB seedlings was performed as follows: the surface of the first to third generations of GeB seedlings was disinfected with 75% alcohol after washing with water, the internal tissues of the GeB seedlings were cut into 1 cm × 1 cm tissue blocks with a sterilization knife, and evenly placed on Potato Dextrose Agar (PDA) plates; the culture was continued after inverting the petri dish after 4 h at 28°C.

### 2.6 Purification of microorganisms

Twenty microliters of each dilution of soil was added to a petri dish containing PDA and incubated at 28°C for 4 h. Each dish was inverted, and incubation was continued. After mycelial growth at the edge of the tissue, the edge of the colony was sampled using apical purification and added to fresh PDA. Four to five rounds of purification were conducted, and, then, a single-culture isolate was obtained.

Cut GeB seedlings were placed on PDA and incubated. The aforementioned apical purification method and procedures were used to obtain a single-culture isolate.

### 2.7 Molecular biological characterization of GeB seedlings and their surrounding soil microorganisms

The cetyltrimethylammonium bromide method was used to extract DNA from the purified and cultured bacterial suspensions by using bacterial and fungi DNA extraction kits (Solarbio). The extracted DNA was purified and stored at −20°C until required. PCR amplification of the ITS region of the fungi and the 16S rRNA gene of the bacterium was performed using universal primers (**Table 2**). The amplified products were detected using 1% agarose gel electrophoresis, and specific bands were detected using a gel imaging system. The PCR products were stored at 4°C and sent to Jilin Comate Bioscience Co. for sequencing. The PCR reaction system comprised 12.5 μL of Taq MasterMix for PAGE, 1.0 μL each of upstream and downstream primers (10 μmol/L), 1.0μL of DNA template, and 9.5μL of deionized water. The PCR reaction conditions involved 30 cycles of pre-denaturation at 94°C for 5 min, denaturation at 94°C for 30 s, annealing at 58°C/54°C for 30 s, and extension at 72°C for 90 s, followed by a final extension at 72°C for 10 min.

**Table 2:** PCR amplification sequence.

Primers	GeB seedlings and their surrounding soil	
	ITS	16S
Forward primers	TCCTCCGCTTATTGATATGC	AGAGTTTGATCCTGGCTCAG
Reverse primers	GGAAGTAAAAGTCGTAACAAGG	TACGGCTACCTTGTTACGACTT

The sequenced base sequences were submitted to the National Center for Biotechnology Information (NCBI) database for a BLAST homology comparison to identify the strains with the highest similarity to each bacterium and determine their biological taxonomic status.

### 2.8 GeB seedlings and their surrounding soil pathogen proving test

The next aim was to prove the disease-inducing effect of three pathogenic bacteria of GeB seedlings. GeB seedlings of the same origin were rinsed with tap water to remove surface soil and other dirt and then wiped with 75% alcohol. By using the spray inoculation method, 10 μL of 10^3^-fold diluted *Trichoderma harzianum*, *Penicillium viridicatum*, and *Fusarium oxysporum* suspension that contained the spores of the fungi on the surface of GeB seedlings was applied. Each treatment was repeated thrice and finally placed into a sterilized self-sealing bag and incubated at 28°C in the incubator. The same amount of sterile water was sprayed on the surface of healthy GeB seedlings alone as a control. Changes in the surfaces of the GeB seedlings were monitored every 3 days. The curative effects of the two pathogens on GeB seedlings were compared after 2 weeks.

### 2.9 Screening of GeB seedlings and their microorganisms for antagonistic strains of soil pathogens

The beneficial bacteria *Pseudomonas* sp. and *Novosphingobium* sp. were isolated and identified as described in Section 2.7. The beneficial bacteria *Paenibacillus polymyxa* (deposited at the China General Microbiological Culture Collection Center, CGMCC: No. 7250) and *Germinating strain* [*Germinating strain D* (JQ364945.1) and *Germinating strain S* (JQ364945.1)] were isolated and preserved in our laboratory and were selected and identified 2.7 isolated and identified single colonies of the GeB pathogens *Trichoderma harzianum*, *Penicillium viridicatum*, and *Fusarium oxysporum*. These were incubated at 28°C overnight in PDB medium; these absorbed 200 μL of spore suspension containing bacteria and fungi and were coated in PDA medium for 3~5 days; and a piece of the PDA was taken from the plate, where the bacteria and fungi grew and the mushrooms cake, and was seeded onto the side of the PDA petri dish. Forty-five PDA mediums were collected, and each PDA medium was treated with one pathogen and one probiotic for confrontation culture. Three replicates were set for each treatment, and the colony confrontation growth of two pathogenic bacteria on GeB seedlings after 1 weeks was compared.

### 2.10 Effect of probiotics on seedling planting

Fungi and bacterial suspensions were prepared as described in Section 2.9. The planting location was the same as that described in Section 2.1, and the continuous cropping farmland planted with GeB in the previous year was selected.

The effect of probiotics on the planting of asexual GeB seedlings was investigated. A method for cultivating GeB in a fungi bag was adopted. Four holes were punched into both sides of each fungi bag filled with *Armillariella mellea*, and 2 mL of beneficial bacteria liquid was inoculated into each well. Different bags were inoculated with different beneficial bacteria. Sterile water was used as a control, and three replicates were used for each treatment. One asexually propagated GeB seedlings was placed in each well, and each bag contains eight GeB seedlings and weighed a total of 116 g. Plants were sown in May and harvested in October. The production performance (weight, quantity, and infection rate) and pharmacodynamic properties of GeB were determined. The determination of pharmacodynamic components was based on a method in Wu et al. (Wu et al., 2022). The effects of beneficial bacteria on planting sexually propagated GeB seedlings were also investigated. Except for the eight sexually propagated GeB seedlings in each bag, the total of 63.6 g differed, and the other methods were the same as those aforementioned.

## 3 Results

### 3.1 Sequence analysis of microbial diversity in GeB seedlings and their surrounding soil

As shown in **Supplementary Table A1**, the percentage of effective sequences for the fungi assay in samples Y, T, and W exceeded 92%, 86%, and 90%, respectively. The percentages of effective sequences for the bacterial assay exceeded 89%, 92%, and 84%, respectively. In samples A, B, and C, the proportion of effective sequences for the fungi assay exceeded 88%, 82%, and 88%, respectively, and the proportion of effective sequences for the bacterial assay exceeded 94%, 93%, and 93%, respectively. Chimeric sequences accounted for less than 10% of the sequences. The high percentage of valid sequences indicated that the sequences obtained from this high-throughput sequencing were suitable for subsequent examination of the interactions between the microbial community structure in GeB seedlings and their surrounding soil.

**Figure 2:**
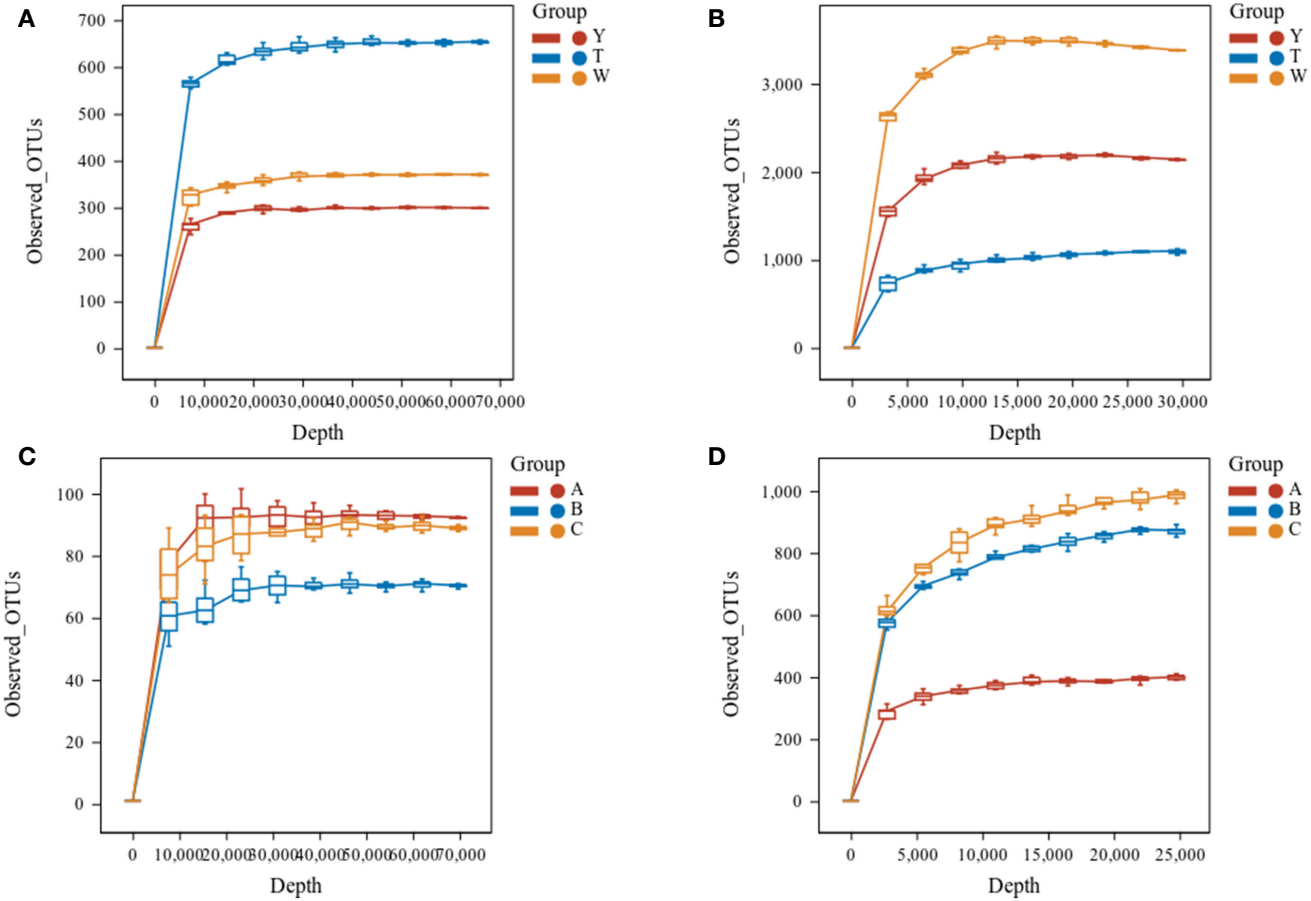
Sparse curves of soil alpha diversity index between the first and third generations of seedlings and their periphery. In a box-and-whisker plot, the meaning of each symbol is as follows: the upper and lower end lines of the box, the upper and lower quartiles [interquartile range (IQR)]; the median line, the median; upper and lower edges, maximum and minimum (extreme values within 1.5× IQR range). **(A)** Rarefaction curves of soil fungi. **(B)** Rarefaction curves of soil bacteria. **(C)** Sparse curve of tuber fungi. **(D)** Sparse curve of tuber bacteria.

The sparse curves of fungi and bacteria in GeB seedlings and their surrounding soil from different generations of propagation were further analyzed (**Figure 2**). The curves tended to flatten when the number of fungi sequences was greater than 30,000, and the number of bacterial sequences was greater than 25,000. The findings indicate that the depth of sequencing could reflect the species information in the samples. The results of species annotation are presented in **Table 3**. A total of 111 phyla, 104 classes, 247 orders, 130 families, and 480 genera were annotated for fungi. A total of 46 phyla, 372 classes, 1021 orders, 3806 families, and 12,327 genera were annotated for the bacteria in the surrounding soil samples with different propagation generations. A total of 10 phyla, 15 classes, 53 orders, 25 families, and 153 genera were annotated for fungi, and 121 phyla, 444 classes, 194 orders, 1,580 families, and 2,983 genera were annotated for bacteria in GeB seedlings samples with different propagation generations.

**Table 3:** Species taxonomic notes.

(A)										
ID	ITS					16S				
	Phylum	Class	Order	Family	Genus	Phylum	Class	Order	Family	Genus
Y1	7	7	16	9	34	3	52	25	465	1,275
Y2	11	4	12	17	33	2	30	36	410	1,169
Y3	14	9	16	10	48	5	47	36	571	1,412
T1	15	11	28	16	58	14	56	307	665	2,357
T2	18	19	63	18	87	8	60	215	736	1,916
T3	21	20	51	19	98	13	70	257	647	2,189
W1	4	7	14	11	34	0	17	45	97	618
W2	12	11	25	12	43	1	19	47	106	716
W3	9	16	22	18	45	0	21	53	109	675
(B)										
ID	ITS					16S				
	Phylum	Class	Order	Family	Genus	Phylum	Class	Order	Family	Genus
A1	1	3	3	2	21	4	40	7	149	226
A2	0	2	6	2	11	7	34	4	113	248
A3	2	3	8	5	22	2	2	4	46	71
B1	0	1	5	1	14	13	83	32	187	441
B2	1	0	6	4	11	9	33	13	112	216
B3	2	1	4	2	19	25	87	47	348	423
C1	0	1	8	2	19	18	48	20	160	404
C2	4	2	6	4	19	24	48	32	188	404
C3	0	2	7	3	17	19	69	35	277	550

### 3.2 Statistical analysis of OTUs of GeB seedlings and their surrounding soil microbial communities

The Venn diagram visualizes the differences and overlaps in the OTUs of fungi and bacterial communities among the samples. The more shared the OTUs, the greater the similarity between the two communities.

**Figure 3:**
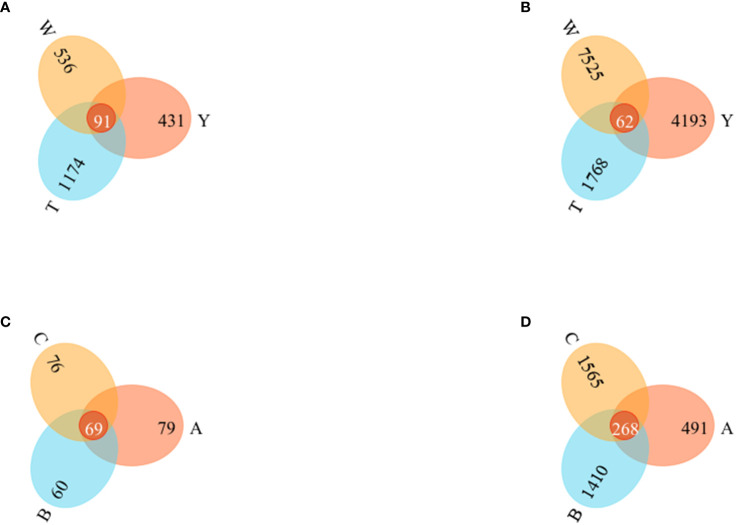
Operational Taxonomic Units (OTUs) Venn diagrams of seedlings from the first to third generations and their surrounding soil. **(A)** OTUs Venn diagram of soil fungi. **(B)** OTUs Venn diagram of soil bacteria. **(C)** OTUS Venn diagram of tuber fungi. **(D)** OTUs Venn diagram of tuber bacteria.

The number of species shared by the fungi communities in the surrounding soils of the nine groups of GeB seedlings from different propagation generations was 91. The number of endemic OTUs in each group was 431 (Y), 1,174 (T), and 536 (W). The endemic OTUs ranked as T>W>Y. Shared OTUs comprised 4.2% of the total OTUs. The number of species shared by the bacterial communities was 62. The number of endemic OTUs in each group was 4193 (Y), 1768 (T), and 7525 (W). The endemic OTUs were ranked W>Y>T. Shared bacterial OTUs accounted for 0.4% of the total OTUs (**Figures 3A, B**).

The number of species common to the fungi community in the nine groups of GeB seedlings from different reproductive generations was 69. The number of unique OTUs in each group was 79 (A), 60 (B), and 76 (C). The unique OTUs were ranked A>C>B. Shared OTUs comprised 4.2% of the total OTUs. The number of species common among the bacterial communities was 268. The number of unique OTUs in each group was 491 (A), 1,410 (B), and 1,565 (C). Unique OTUs were ranked C>B>A. The shared OTUs comprised 0.4% of the total OTUs.

The collective findings indicate that the structure of bacteria and fungi changed significantly after different generations of breeding, but the similarity of microbial and fungi communities was greater in GeB seedlings than in the other groups (**Figures 3C, D**).

### 3.3 Alpha diversity analysis of GeB seedlings and their surrounding soil

The microbial communities in the surrounding soil of the GeB seedlings and GeB seedlings samples were analyzed for alpha diversity by using a statistical t-test. Chao1 and the Observed species indices were used to indicate richness, with higher values representing higher species richness in the microbial community. The Pielou’s evenness index (Pieloue) was used to represent community evenness, with higher values indicating higher species evenness in the microbial community. Shannon and Simpson indices were used to represent community diversity, with higher values indicating greater species diversity in the microbial community.

In the periphyton soil fungi community of GeB seedlings in sample Y, Chao1, Observed species, Pieloue, Shannon, and Simpson indices were lower than that of samples W and T, indicating that uniformity and richness were lowest, and community diversity was less in the reproduction generation. In the bacterial community of sample W, Chao1, Observed species, Pieloue, Shannon, and Simpson indices were significantly higher than that of samples Y and T (P < 0.05), indicating the highest homogeneity and abundance, richer community diversity in reproduction generation three was also observed (**Tables 4A, B**).

**Table 4:** Community diversity index.

(A)					
Group	Chao1	Observed species	Pieloue	Shannon	Simpson
Y	299.12 ± 16.8b	297.63 ± 17.11b	0.46 ± 0.02b	3.8 ± 0.17b	0.74 ± 0.04b
T	653.11 ± 113.44a	643.53 ± 108.47a	0.73 ± 0.02a	6.77 ± 0.01a	0.97 ± 0a
W	370.24 ± 62.56b	366.27 ± 62.94b	0.49 ± 0.07b	4.21 ± 0.69b	0.8 ± 0.08ab
(B)					
Group	Chao1	Observed species	Pieloue	Shannon	Simpson
Y	2138.59 ± 152.73b	2038.7 ± 130.98b	0.79 ± 0.01b	8.64 ± 0.05b	0.98 ± 0b
T	1090.18 ± 33.68c	905.43 ± 24.22c	0.33 ± 0c	3.21 ± 0.03c	0.66 ± 0c
W	3381.96 ± 142.7a	3306.63 ± 119.25a	0.89 ± 0.01a	10.39 ± 0.12a	1 ± 0a
(C)					
Group	Chao1	Observed species	Pieloue	Shannon	Simpson
A	87.06 ± 16.3a	86.83 ± 16.24a	0.31 ± 0.02a	1.94 ± 0.14a	0.69 ± 0.02a
B	72.56 ± 6.25a	72.2 ± 6.01a	0.38 ± 0.06a	2.34 ± 0.31a	0.74 ± 0.05a
C	91.89 ± 17.05a	90.5 ± 16.51a	0.41 ± 0.05a	2.64 ± 0.24a	0.79 ± 0.04a
(D)					
Group	Chao1	Observed species	Pieloue	Shannon	Simpson
A	399.28 ± 121.91b	363.53 ± 104.06b	0.62 ± 0.05a	5.16 ± 0.12a	0.93 ± 0.02a
B	872.11 ± 195.79ab	763.97 ± 162.93ab	0.59 ± 0.01a	5.64 ± 0.25a	0.92 ± 0.01a
C	988.2 ± 99.59a	842.8 ± 98.74a	0.58 ± 0.03a	5.64 ± 0.35a	0.91 ± 0.02a

Mean ± SD (n= 3). Data with the different letter(s) in the same column are significantly different (P <0.05).

In the fungi community of GeB seedlings of sample C, Chao1, Observed species, Pieloue, Shannon, and Simpson indices were higher than that of samples A and B, indicating that the uniformity, richness, and community diversity were richest in the propagation of the three generations. In the bacterial community of sample C, Chao1, Observed species, and Shannon indices were higher than that of samples A and B, indicating that the richness, diversity, and community diversity were richest in the propagation of the three generations. However, no significant differences were observed (**Tables 4C, D**).

In general, the fungi and bacterial community diversity of the third generation of GeB seedlings and their surrounding soils was richer than those of the second and first generations.

### 3.4 Analysis of the composition of dominant species in GeB seedlings and their surrounding soil

At the genus level, the composition of fungi and bacterial communities varied widely different samples (GeB seedlings and their surrounding soil). The sequencing results showed that the dominant fungi genera in the soil around GeB seedlings were *Pseudogymnoascus* and *Fusarium* (**Supplementary Figure A1A**). The dominant bacterial genera in the soil around the GeB seedlings were *Escherichia-Shigella* and *Rhodanobacter* (**Supplementary Figure A1B**). The dominant fungi genera in the GeB seedlings were *Alternaria* and *Penicillium* (**Supplementary Figure A1C**). The dominant bacterial genera in the GeB seedlings were *Vibrio* and *Caulobacter* (**Supplementary Figure A1D**).

**Figure 4:**
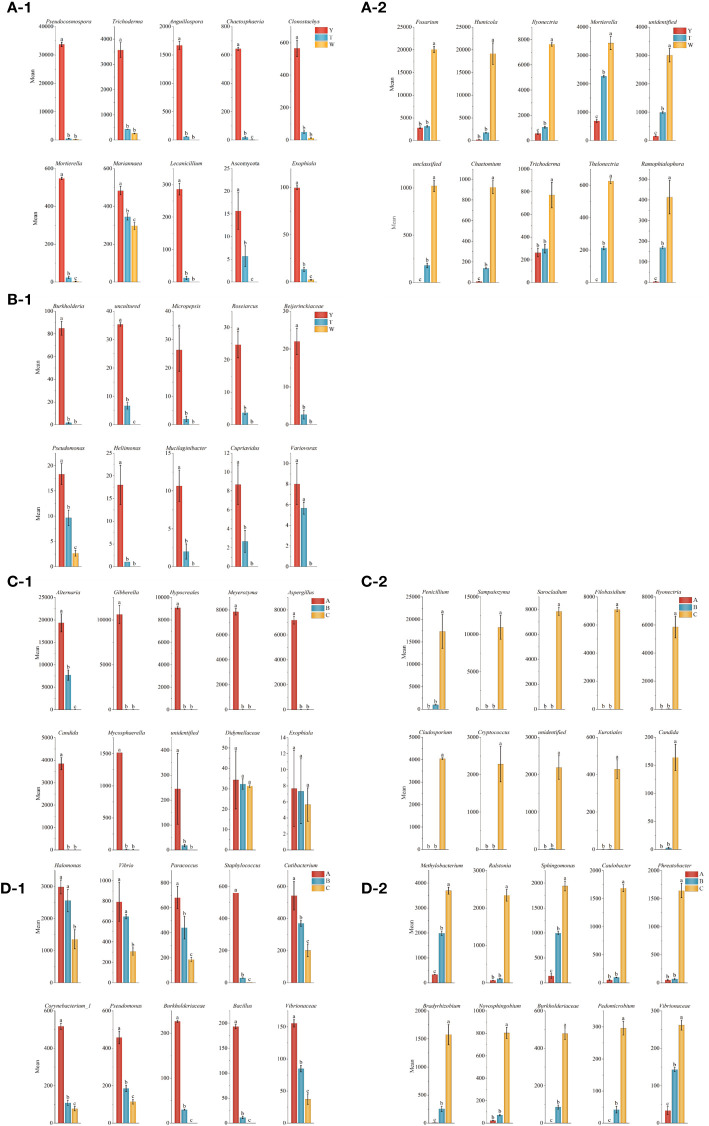
Analysis of dominant strains in the first to third generations of seedlings and their surrounding soil. At the genus level, the top 10 fungi and bacteria in the seedlings of seedlings with three reproductive generations had an increasing and decreasing trend. **(A-1)** Fungi in the soil around the seedlings decreasing with the number of generations. **(A-2)** Fungi in the soil around the seedlings increasing with the number of generations. **(B-1)** Bacteria in the soil around the seedlings decreasing with the number of generations. **(C-1)** Fungi in seedlings decreasing with the number of generations. **(C-2)** Fungi in seedlings increasing with the number of generations. **(D-1)** Bacteria in seedlings decreasing with the number of generations. **(D-2)** Bacteria in seedlings increasing with the number of generations.

A histogram of the top 10 genus level of microorganisms in the periphyton soil of GeB seedlings and the surrounding soil was plotted. As shown in **Figure 4**, among the surrounding soil fungi, the genera that increased with continued generations (**Figure 4A-1**) were *Fusarium*, *Humicola*, *Ilyonectria*, *Mortierella*, *unidentified* and *unclassified fungi*, *Chaetomium*, *Trichoderma*, *Thelonectria*, and *Ramophialophora*. The genera that decreased with continued generation (**Figure 4A-2**) were *Pseudocosmospora*, *Trichoderma*, *Anguillospora*, *Chaetosphaeria*, *Clonostachys*, *Mortierella*, *Mariannaea*, *Lecanicillium*, *Ascomycota*, and *Exophiala.*

Among the surrounding soil bacteria, 91 genera increased in number with continued generations (**Supplementary Table A2**). The genera that decreased with continued generations (**Figure 4B-1**) were *Burkholderia*, *uncultured*, *Micropepsis*, *Roseiarcus*, *Beijerinckiaceae*, *Pseudomonas*, *Heliimonas*, *Mucilaginibacter*, *Cupriavidus*, and *Variovorax*.

Among the GeB seedlings of fungi, genera that increased with the number of generations were *Penicillium*, *Sampaiozyma*, *Sarocladium*, *Filobasidium*, *Ilyonectria*, *Cladosporium*, *Cryptococcus*, *unidentified Eurotiales*, and *Candida* (**Figure 4C-1**). Genera that decreased with continued generations were *Alternaria*, *Gibberella*, *Hypocreales*, *Meyerozyma*, *Aspergillus*, *Candida*, *Mycosphaerella*, *unidentified Didymellaceae*, and *Exophiala* (**Figure 4C-2**).

Among the GeB seedlings of bacteria, genera that increased with continued generations were *Methylobacterium*, *Ralstonia*, *Sphingomonas*, *Caulobacter*, *Phreatobacter*, *Bradyrhizobium*, *Novosphingobium*, *Burkholderiaceae*, *Pedomicrobium*, and *Vibrionaceae* (**Figure 4D-1**). Genera that decreased in number with continued generations were *Halomonas*, *Vibrio*, *Paracoccus*, *Staphylococcus*, *Cutibacterium*, *Corynebacterium_1*, *Pseudomonas*, *Burkholderiaceae*, *Bacillus*, and *Vibrionaceae* (**Figure 4D-2**).

Because GeB seedlings undergo degradation during asexual reproduction, a reasonable inference is that pathogenic bacteria are present in genera that increase with the number of generations, and beneficial bacteria are present in genera that decrease with the number of generations. The presence of *Trichoderma* within the periphyton soil of asparagus was significant (P < 0.05) in all three generations of asexually propagated GeB seedlings. Notably, although *Trichoderma* was present in increasing and decreasing genera, there was a significant difference with the increasing genera, including *T. hamatum* antagonizing phytopathogenic bacteria (Poveda et al., 2023), which can cause soft rot of *Gastrodia* (Poveda et al., 2023), and the decreasing genera, including *T. atroviride*, a plant probiotic that can promote plant health and nutrition (Contreras-Cornejo et al., 2018), increase insect resistance of maize leaves, and inhibit the production of wilt disease in maize crops (Coninck et al., 2020).

In this study, *Fusarium* increased with the number of generations and was speculated to be a pathogenic bacterium. In addition, *Fusarium* can cause black rot and soft rot of GeB (Li et al., 2022), which is consistent with the literature. In GeB seedlings, *Penicillium raperi* increased with increasing generations, and Liu et al. (Liu et al., 2019) found that GeB disease was caused by *P. oxalicum*. Regarding the *Novosphingobium* in this study, its abundance increased with increasing algebra. Studies have shown that *Novosphingobium* can transfer ginsenosides (Kim et al., 2013). The topics of whether the bacteria are beneficial bacterium and antagonistic to other pathogenic bacteria require further research.

The aforementioned findings were also obtained in the immature tuber seedling perimeter soil and GeB seedlings within the common genera *Exophiala*, *Ilyonectria*, and *Pseudomonas*, showing a gradual decrease in *Pseudomonas* and *Exophiala* and a gradual increase in *Ilyonectria*.

### 3.5 Association analysis of GeB seedlings and their surrounding soil dominant species

*Trichoderma*, *Fusarium*, *Ilyonectria*, *Penicillium*, *Sphingomonas*, and *Pseudomonas* were also correlated (**Table 5**). With the increasing number of reproductive generations, there was a significant negative correlation between *Trichoderma*, *Fusarium*, *Ilyonectria*, and *Penicillium* (P < 0.05). *Pseudomonas* showed a significant positive correlation (P < 0.01). *Sphingomonas* was significant negatively correlated with *Pseudomonas* (P < 0.05).

**Table 5:** Microbiota correlation analysis.

	*Trichoderma*	*Fusarium*	*Ilyonectria*	*Penicillium*	*Sphingomonas*	*Pseudomonas*
*Trichoderma*	1					
*Fusarium*	0.975**	1				
*Ilyonectria*	0.965**	0.998**	1			
*Penicillium*	0.926**	0.967**	0.974**	1		
*Sphingomonas*	0.865**	0.884**	0.907**	0.881**	1	
*Pseudomonas*	−0.671*	−0.664	−0.700*	−0.681*	−0.924**	1

*P<0.05; **P<0.01.

### 3.6 Differential species analysis of GeB seedlings and their surrounding soil microbial communities

**Figure 5:**
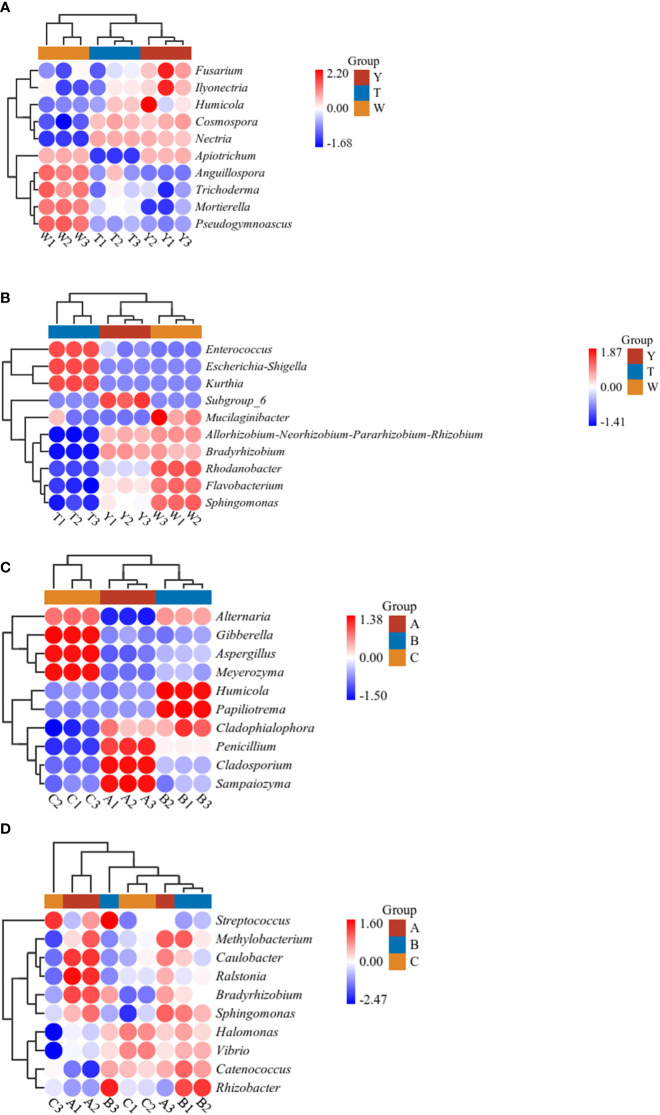
Heat map analysis of soil microbial communities in and around seedlings from the first to third generations. Heat maps are drawn using the heat map tool in the GenesCloud platform (https://www.genescloud.cn). Data were normalized by z-scores, and Euclidean distances were subjected to UPGMA cluster analysis. **(A)** Heat map analysis of soil fungi. **(B)** Heat map analysis of soil bacteria. **(C)** Heat map analysis of tuber fungi. **(D)** Heat map analysis of tuber bacteria.

The analysis of species occupying the top 10 differences in species composition was conducted (**Figure 5**). As shown in the species composition heat map, the genera with significant differences in fungi abundance at the genus level of the soil samples were *Apiotrichum*, *Anguillospora*, *Pseudogymnoascus*, *Trichoderma*, *Humicola*, *Fusarium*, *Ilyonectria*, *Cosmospora*, *Mortierella*, and *Nectria*. The genera with significant differences in bacterial abundance were *Kurthia*, *Escherichia-Shigella*, *Enterococcus*, *Subgroup_6*, *Bradyrhizobium*, *Rhodanobacter*, *Mucilaginibacter*, *Flavobacterium*, *Allorhizobium-Neorhizobium-Pararhizobium-Rhizobium*, and *Sphingomonas*.

At the genus level of the tuber samples, the genera with significant differences in fungi abundance were *Gibberella*, *Sampaiozyma*, *Penicillium*, *Cladosporium*, *Humicola*, *Alternaria*, *Meyerozyma*, *Papiliotrema*, *Aspergillus*, and *Cladophialophora*. The genera showing significant differences in bacterial abundance were *Methylobacterium*, *Sphingomonas*, *Bradyrhizobium*, *Caulobacter*, *Ralstonia*, *Rhizobacter*, *Streptococcus*, *Catenococcus*, *Vibrio*, and *Halomonas*. The heat map results showed that different generations of propagation affected the microbial diversity of GeB and the surrounding soil.

### 3.7 Isolation and characterization of asexually propagated GeB seedlings and their surrounding soil microbial dominant strains

**Figure 6:**
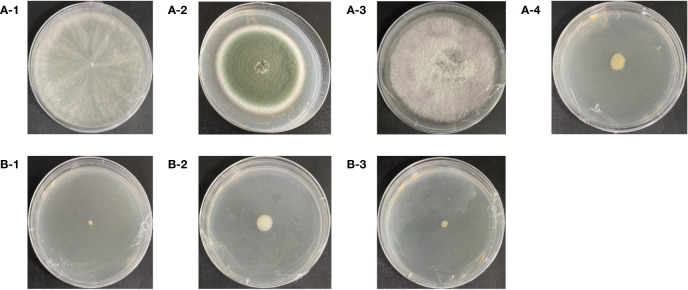
Isolation of soil microorganisms from the first to third generations of seedlings and their surroundings. **(A-1)** *Trichoderma harzianum*. **(A-2)** *Penicillium viridicatum*. **(A-3)** *Fusarium oxysporum*. **(A-4)** *Novosphingobium*. **(B-1)** *Pseudomonas* sp. **(B-2)** *Agrobacterium rhizomes*. **(B-3)** *Herbaspirillum hiltneri*.

Three fungi strains and one bacterial strain were isolated and identified from the surrounding soil of the third generations of propagated GeB. The strains are shown in **Figure 6A** with base peak maps (**Supplementary Figures A2A–D**) and sequences (**Supplementary Figures A3A–D**). The sequence length of this strain was more than 99% homological to the registered genes in the NCBI database. More than 96% coverage of the same base (MT422092.1, MK583349.1, OP881816.1, and KM252978.1, respectively) identifies the fungi as *T. harzianum* (OR518627.1), *P. viridicatum* (OR518628.1), and *F. oxysporum* (OR511432.1) and the bacteria as *Novosphingobium* (OR529195.1).

Three bacteria strains were isolated and identified from the surrounding soil the first generation of propagated GeB. The resulting strains are shown in **Figure 6**, with the base peak maps (**Supplementary Figures A2E–G**) and sequences (**Supplementary Figures A3E–G**). The sequence length of this strain was more than 99% homological to the registered genes in the NCBI database. More than 99% coverage of the same base (AP021904.1, EU420078.1, and KU305712.1, respectively), identifies the bacteria as *Pseudomonas* sp. (OR501395.1), *Agrobacterium rhizomes* (OR501394.1), and *Herbaspirillum hiltneri* (OR588778.1).

### 3.8 Pathogen strain splice back test

**Figure 7:**
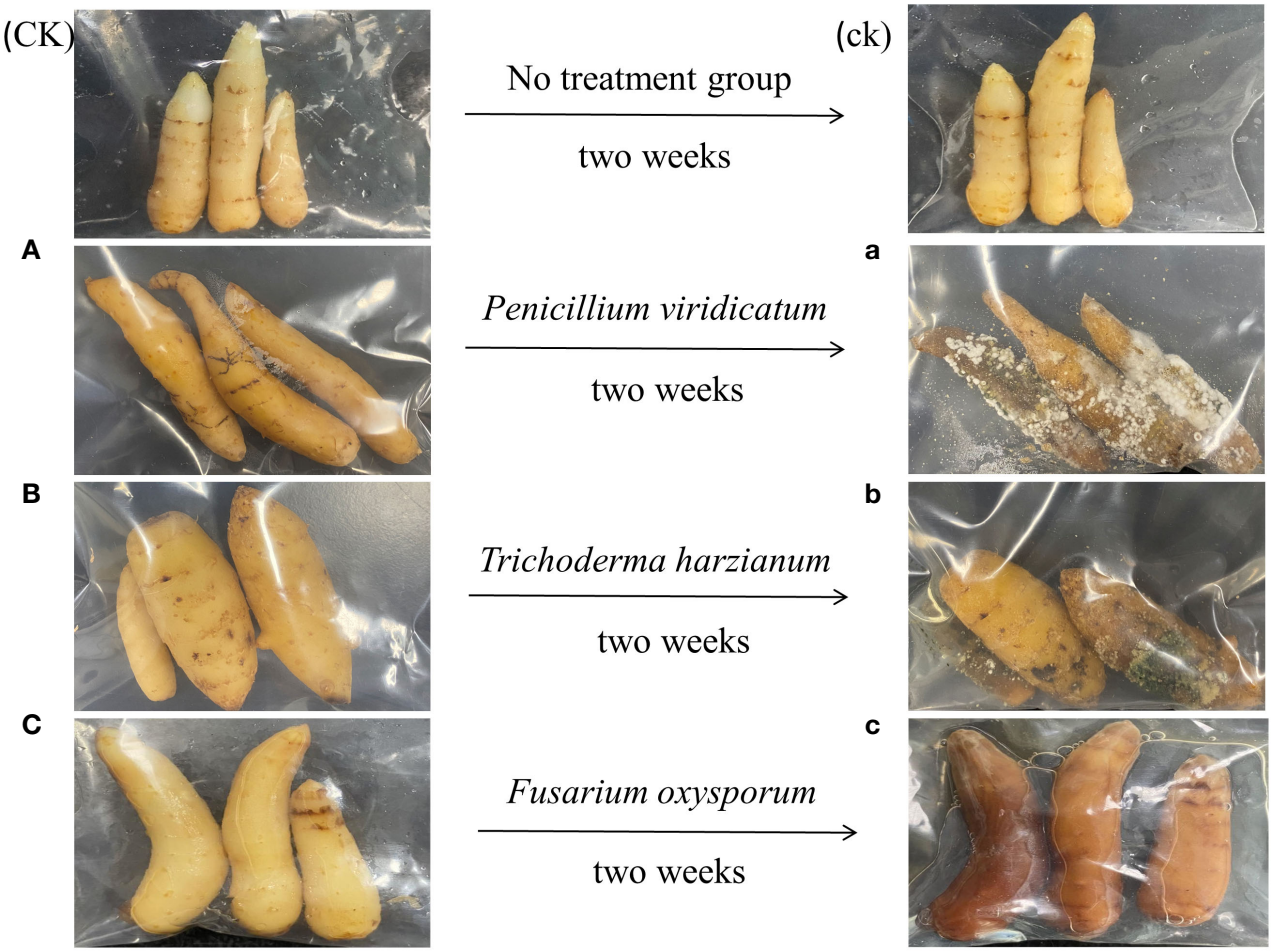
Pathogenic fungi back-grafting verification for isolation and identification of seedlings from the first to third generations and their surrounding soil. On the left is the tuber infected before treatment, and on the right is the tuber 2 weeks after spraying the pathogen. CK, control group sprayed with sterile water; ck, control group after spraying sterile water for 2 weeks. **(A)** Seedlings that have not been sprayed with *Penicillium viridicatum*; (a) immature tuber seedlings 2 weeks after spraying *Penicillium viridicatum*. **(B)** Seedlings that have not been sprayed with *Trichoderma harzianum*; (b) seedlings 2 weeks after spraying *Trichoderma harzianum*. **(C)** Seedlings that have not been sprayed with *Fusarium oxysporum*; (c) seedlings 2 weeks after spraying *Fusarium oxysporum*.

The pathogenicity of three pathogenic fungi (*P. viridicatum*, *T. harzianum*, and *F. oxysporum*) in GeB seedlings is shown in **Figure 7**. When sterile water was sprayed as a control, lesions occurred on the surface of GeB seedlings within 14 days. After spraying the isolated and identified pure *P. viridicatum*, many brown plaques were formed in a relatively short period of time. After 14 days, a white fluffy mold appeared on the surface of the GeB seedlings, and the white hyphae in the middle of the white hyphae appeared in the later stage, and the GeB seedlings wilted. These results are consistent with the characteristics of pure *P. viridicatum* infection, further proving that the strain was pure *P. viridicatum*. After spraying the isolated and identified pure *T. harzianum*, a small amount of white hyphae formed on the surface of the GeB seedlings within a relatively short period. The edges of the white hyphae gradually changed from white to yellow and finally to green. After 14 days, the GeB seedlings became moist and soft, consistent with the characteristics of *T. harzianum*, which further proved that the strain was *T. harzianum*. After spraying *F. oxysporum*, a small number of white hyphae appeared on the surface of the GeB seedlings. After 14 days, the surface of the white mycelia turned brown with a small amount of sticky substance, the white fluffy mycelia disappeared, and the surface of the GeB seedlings was soft and turned red. These characteristics are consistent with those of *F. oxysporum* infection, further confirming that the strain was *F. oxysporum*.

In general, compared with the control group, the GeB seedlings of GeB seedlings in the experimental group had different degrees of decay after spraying the three pathogenic bacteria, but the decay and pathogenicity of *P. viridicatum* were higher than those of the other two pathogens.

### 3.9 Screening of GeB seedlings and their periphyton for antagonistic strains of soil pathogens

**Figure 8:**
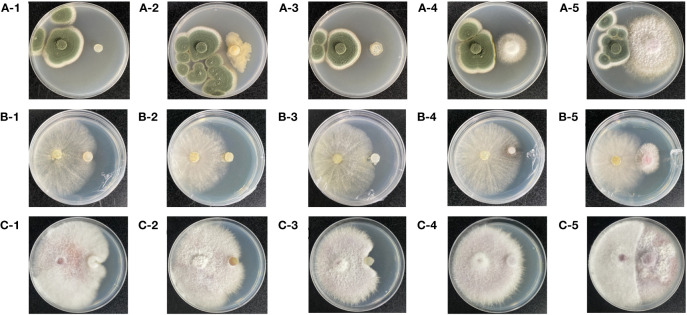
The first to third generations of seedlings and their surrounding soil pathogens and beneficial bacteria were cultured. Observe the growth of colonies and the presence of inhibitory boundaries after 1 week. Pathogenic fungi on the left and beneficial fungi and bacteria on the right. **(A-1)** *Penicillium viridicatum* and *Pseudomonas* sp. **(A-2)** *Penicillium viridicatum* and *Novosphingobium*. **(A-3)** *Penicillium viridicatum* and *Paenibacillus polymyxa*. **(A-4)** *Penicillium viridicatum* and *Germinating strain D.* **(A-5)** *Penicillium viridicatum* and *Germinating strain S*. **(B-1)** *Trichoderma harzianum* and *Pseudomonas* sp. **(B-2)** *Trichoderma harzianum* and *Novosphingobium*. **(B-3)** *Trichoderma harzianum* and *Paenibacillus polymyxa*. **(B-4)** *Trichoderma harzianum* and *Germinating strain D.* **(B-5)** *Trichoderma harzianum* and *Germinating strain S*. **(C-1)** *Fusarium oxysporum* and *Pseudomonas* sp. **(C-2)** *Fusarium oxysporum* and *Novosphingobium*. **(C-3)** *Fusarium oxysporum* and *Paenibacillus polymyxa*. **(C-4)** *Fusarium oxysporum* and *Germinating strain D.* **(C-5)** *Fusarium oxysporum* and *Germinating strain S*.

The results of the confrontation assays of three pathogenic fungi (*P. viridicatum*, *T. harzianum*, and *F. oxysporum*) and five strains of beneficial bacteria (*Pseudomonas* sp., *Novosphingobium* sp., *Paenibacillus polymyxa*, *Germinating strain D*, and *Germinating strain S*) are shown in **Figure 8**. There was an antagonistic relationship between the isolated and identified pure *P. viridicatum* and *Germinating bacteria D* (**Figure 8A**). However, there was no obvious antagonistic effect on *Pseudomonas* sp., *Novosphingobium* sp., and *Paenibacillus polymyxa*. *Germinating bacteria S*. *T. harzianum* has an antagonistic effect against *Paenibacillus polymyxa* and *Germinating bacteria S* (**Figure 8B**). However, there was no obvious antagonistic effect on *Pseudomonas* sp. and *Novosphingobium* sp. *Germinating bacteria D*. *F. oxysporum* has an antagonistic effect with *Paenibacillus polymyxa* and *Germinating bacteria S* (**Figure 8C**). However, there was no obvious antagonistic effect with *Pseudomonas* sp., *Novosphingobium* sp., and *Germinating bacteria D*.

### 3.10 Effects of probiotic fungi and bacteria on the production performance of GeB in seedlings planting

Treatment of the first generation of GeB seedlings with five probiotic fungi and bacteria with first generation of GeB seedlings had a significant effect on the agronomic traits and infection rate of GeB (P < 0.05; **Supplementary Table A3A**). *Novosphingobium* sp. and *Pseudomonas* sp. significantly increased the weight of GeB (P < 0.05). However, there was no significant difference in the weight of GeB between *Paenibacillus polymyxa*, *Germinating strain D*, and *Germinating strain S* (P > 0.05). *Novosphingobium* sp. and *Pseudomonas* sp. significantly increased the number of GeB (P < 0.05). There was no significant difference in the number of GeB between *Paenibacillus polymyxa*, *Germinating strain D*, and *Germinating strain S* compared with that in the control group (P > 0.05). *Germinating strain S* increased the GeB infection rate significantly (P < 0.05), and there was no significant difference in the infection rate between the other strains and the control group (P > 0.05). Five strains of probiotic fungi-treated and bacteria-treated first generation of GeB seedlings had significant effects on the planting of the six medicinal components of GeB (**Supplementary Figure A4**; **Supplementary Table A4A**). The order of gastrodin content from high to low was *Germinating strain S* > *Germinating strain D* > *Paenibacillus polymyxa* > *Novosphingobium* sp. > CK > *Pseudomonas* sp. The order of 4-Hydroxybenzyl alcohol content from high to low was *Paenibacillus polymyxa* > CK > *Germinating strain S* > *Pseudomonas* sp. > *Germinating strain D* > *Novosphingobium* sp. The order of Parishin E content from high to low is *Germinating strain S* > *Germinating strain D* > CK > *Novosphingobium* sp. > *Paenibacillus polymyxa* > *Pseudomonas* sp. The order of Parishin B content from high to low was *Germinating strain S* > *Germinating strain D* > CK > *Pseudomonas* sp. > *Novosphingobium* sp. > *Paenibacillus polymyxa.* The order of Parishin C content from high to low was *Germinating strain S* > *Germinating strain D* > *Paenibacillus polymyxa* > *Pseudomonas* sp. > CK > *Novosphingobium* sp. The order of Parishin A content from high to low was *Germinating strain S* > *Germinating strain D* > *Pseudomonas* sp. > *Novosphingobium* sp. > *Paenibacillus polymyxa* > CK.

Treatment of the third generation of GeB seedlings with five probiotic fungi and bacteria with the third generation of GeB seedlings had a significant effect on the agronomic traits and infection rate of GeB (P < 0.05; **Supplementary Table A3B**). *Pseudomonas* sp., *Novosphingobium* sp., and *Germinating strain S* significantly increased the weight of GeB (P < 0.05). However, there was no significant difference in the weight of GeB between *Germinating strain D* and *Paenibacillus polymyxa* compared with that of CK (P > 0.05). There was no significant difference in the number of GeB between the strains and the CK (P > 0.05). *Pseudomonas* sp. significantly reduced the GeB infection rate (P < 0.05), and there was no significant difference in the infection rate between other strains and the CK (P > 0.05). Five strains of probiotic fungi-treated and bacteria-treated third generation of GeB seedlings had significant effects on the planting of six medicinal components of GeB (**Supplementary Figure A5**; **Supplementary Table A4B**). The order of gastrodin content from high to low was *Germinating strain S* > *Pseudomonas* sp. > *Germinating strain D* > *Paenibacillus polymyxa* > *Novosphingobium* sp. > CK. The order of 4-Hydroxybenzyl alcohol content from high to low was *Germinating strain D* > *Germinating strain S* > *Novosphingobium* sp. > *Pseudomonas* sp. > CK > *Paenibacillus polymyxa.* The order of Parishin E content from high to low was *Pseudomonas* sp. > *Germinating strain S* > *Germinating strain D* > *Paenibacillus polymyxa* > *Novosphingobium* sp. > CK. The order of Parishin B content from high to low was *Germinating strain S* > *Pseudomonas* sp. > *Germinating strain D* > *Novosphingobium* sp. > *Paenibacillus polymyxa* > CK. The order of Parishin C content from high to low was *Germinating strain S* > *Pseudomonas* sp. > *Germinating strain D* > *Novosphingobium* sp. > *Paenibacillus polymyxa* > CK. The order of Parishin A content from high to low was *Germinating strain S* > *Pseudomonas* sp. > *Germinating strain D* > *Paenibacillus polymyxa* > *Novosphingobium* sp. > CK.

## 4 Discussion

The asexual propagation of GeB seedlings was seriously degraded (Xu and Guo, 2000). However, the causes of the degradation of multi-generation asexually reproduced GeB seedlings have not yet been clarified. More than 99% of orchids are partially dependent on symbiotic mycorrhizae, and there is another type of orchid that needs to rely entirely on mycorrhizal nutrition for its entire life cycle (Suetsugu et al., 2017). GeB relies entirely on mycorrhizal nutrition heterotrophic plant of the genus Orchidaceae, which is completely dependent on *Mycena* and *Armillariella mellea* for its entire life cycle. The degradation of GeB seedlings reproduced asexually mainly manifested as severe diseases, and the degradation of Orchidaceae has not been reported.

This study is the first to investigate the causes of degradation during the multi-generational propagation of GeB from a microbial perspective. Because GeB has problems such as reduced yield and variety degradation with the increase of generations, it relies on fungi to provide nutrients during the growth of GeB. Therefore, we speculate that the changes in microorganisms are causing their degeneration. The results showed that the bacterial and fungal composition of GeB seedlings and the surrounding soil changed significantly with different propagation generations, which was consistent with our assumptions (**Supplementary Figure A1**). The alpha diversity analysis demonstrated that the community diversity of fungi and bacteria in the second and third generations of GeB seedlings and the surrounding soil was richer than that of the first generation. In addition, Sections 3.4 and 3.7 showed that there were more pathogenic fungi and bacteria in the third generation of GeB seedlings and the surrounding soil than that in the first and second generations. The increase in the species and number of pathogenic fungi and bacteria may have led to the generational degradation of GeB.

*Trichoderma* was found in the asexual reproduction GeB seedlings and the surrounding soil, which increased and decreased with the number of reproductive generations, but did not belong to the same species. The increasing strains are *T. hamatum* causing GeB soft rot (López López et al., 2022; Han M. et al., 2017); the reduced strains were *T. atroviride*, which increased the insect resistance of maize leaves and inhibited the production of wilt disease in maize crops (Coninck et al., 2020), which was speculated to be a probiotic of GeB seedlings. At the same time, it has been reported that *Fusarium* (Li et al., 2022), *Ilyonectria* (Qiao et al., 2019), and *Penicillium* (Liu et al., 2019) are all pathogenic bacteria of GeB. In this study, these three strains increased with the increase of generations, which verified that they were pathogenic bacteria of GeB, which was consistent with previous studies. *Pseudomonas* (Adhikari et al., 2001; Kuklinsky-Sobral et al., 2004; Preston et al., 2004) can effectively control phytohormones and the solubilization of minerals, and it is speculated to be a probiotic of GeB. In this study, it decreased with the increase of reproductive generations, and it was the dominant bacterium in the first generation of healthy GeB, which was verified to be a probiotic for GeB seedlings. *Sphingomonas* (Li et al., 2019) can symbiosis with plants and can play a role in antagonizing plant pathogenic fungi and promoting plant growth, which increases with the increase of reproductive generations (Mazoyon et al., 2023). However, further in-depth research is required to determine whether this is probiotic. In this study, it was concluded that the decrease of beneficial fungi and the increase of pathogenic fungi were the reasons for the degradation of asexual reproduction of GeB seedlings, and it was speculated that the decrease of beneficial fungi may be the reason for the inhibition of pathogenic fungi.

The medicinal plant endophytes that can reduce and prevent the harmful effects of a wide range of pathogens are (Pageni et al., 2014), for example, *Cellulomonas marina* (Yan et al., 2020), *Amycolatopsis* (Narsing Rao et al., 2020), *Paracoccus endophyticus* sp. (Zhang et al., 2019), *Pseudomonas* sp. (Li et al., 2018), and *Sphingomonas mesophila* sp. (Li et al., 2019). *Pseudomonas* and *Novosphingobium* were isolated in this study. In addition, three fungi strains (*T. harzianum*, *P. viridicatum*, and *F. oxysporum*) and two bacterial strains (*A. rhizomes* and *H. hiltneri*) were obtained. These microbes may be valuable in overcoming the degradation of multi-generational asexually propagated GeB seedlings. Amplicon sequencing showed that *Penicillium* was relatively abundant at the genus level in GeB seedlings. However, *Penicillium viridicatum* was isolated from the soil around the GeB seedlings. Therefore, the analysis may have used *Penicillium* sp. as a common strain of GeB seedlings and perisoil microorganisms. In addition, backlash experiments proved that *Penicillium viridicatum* can cause serious diseases in GeB seedlings and have speculated that *Penicillium* sp. is an important genus for GeB degeneration. This phenomenon proves that soil microorganisms and plant growth are inseparable (de-Bashan et al., 2020).

According to a review of the literature, there have been no report on the antagonistic bacteria and fungi of the pathogenic fungi of vegetatively propagated GeB seedlings; thus, determining the antagonistic bacteria and fungi of three multi-generation vegetatively propagated GeB seedlings would fill a gap in the literature. In this study, five beneficial bacteria were selected for pathogenic fungi antagonism.

The results showed that the GeB seedlings pathogen *P. viridicatum* was antagonized by *Germinating strain D* and *P. polymyxa*, *Germinating strain S* antagonized by *T. harzianum*, and *P. polymyxa* and *Germinating strain S* antagonized by *F. oxysporum* (**Figure 8**). Therefore, we speculated that supplementation with *Pseudomonas* sp., *Novosphingobium* sp., *Germinating strain S*, *Germinating strain D*, and *P. polymyxa* reduces the degradation of asexual GeB seedlings and increases the yield of GeB.

## 5 Conclusions

A possible reason for the degradation of asexual reproduction by *G. elata Bl. f. glauca S*. chow (GeB) on Changbai Mountain was a combined effect of increased pathogenic bacteria, such as *P. viridicatum*, and decreased beneficial bacteria, such as *Pseudomonas.* Three strains of *P. viridicatum* and other pathogenic bacteria were isolated and identified from third generations of asexually propagated GeB seedlings and the soil. Four strains of *Pseudomonas* and other beneficial bacteria were isolated and identified from first generation of asexually propagated GeB and the surrounding soil. The antagonist of the asexual reproductive degradation pathogen *P.viridiatum* is *Emergence Bacterium D*. The antagonists of the asexual reproduction degradation pathogens *T. harzianum* and *F. oxysporum* are *P. polymyxa* and *Emergence Bacterium S*. In conclusion, beneficial fungi and bacteria reduce the degradation of the asexual reproduction of GeB seedlings.

## Data availability statement

The datasets presented in this study can be found in online repositories. The names of the repository/repositories and accession number(s) can be found below: GenBank OR511432, OR501395, OR501394, OR518628, OR518627, OR529195, OR588778.

## Author contributions

XW: Data curation, Investigation, Methodology, Writing – original draft, Writing – review & editing. YG: Funding acquisition, Supervision, Writing – review & editing. PZ: Investigation, Writing – review & editing. GZ: Investigation, Writing – review & editing. XY: Investigation, Writing – review & editing. QL: Investigation, Writing – review & editing.
