# Supplementary figures and images for: Decrease in beneficial bacteria and increase in harmful bacteria in Gastrodia seedlings and their surrounding soil are mainly responsible for degradation of Gastrodia asexual propagation
Source: Front Plant Sci. 2024 Feb 6;15:1334958. doi: 10.3389/fpls.2024.1334958 (PMC10877603; doi:10.3389/fpls.2024.1334958)

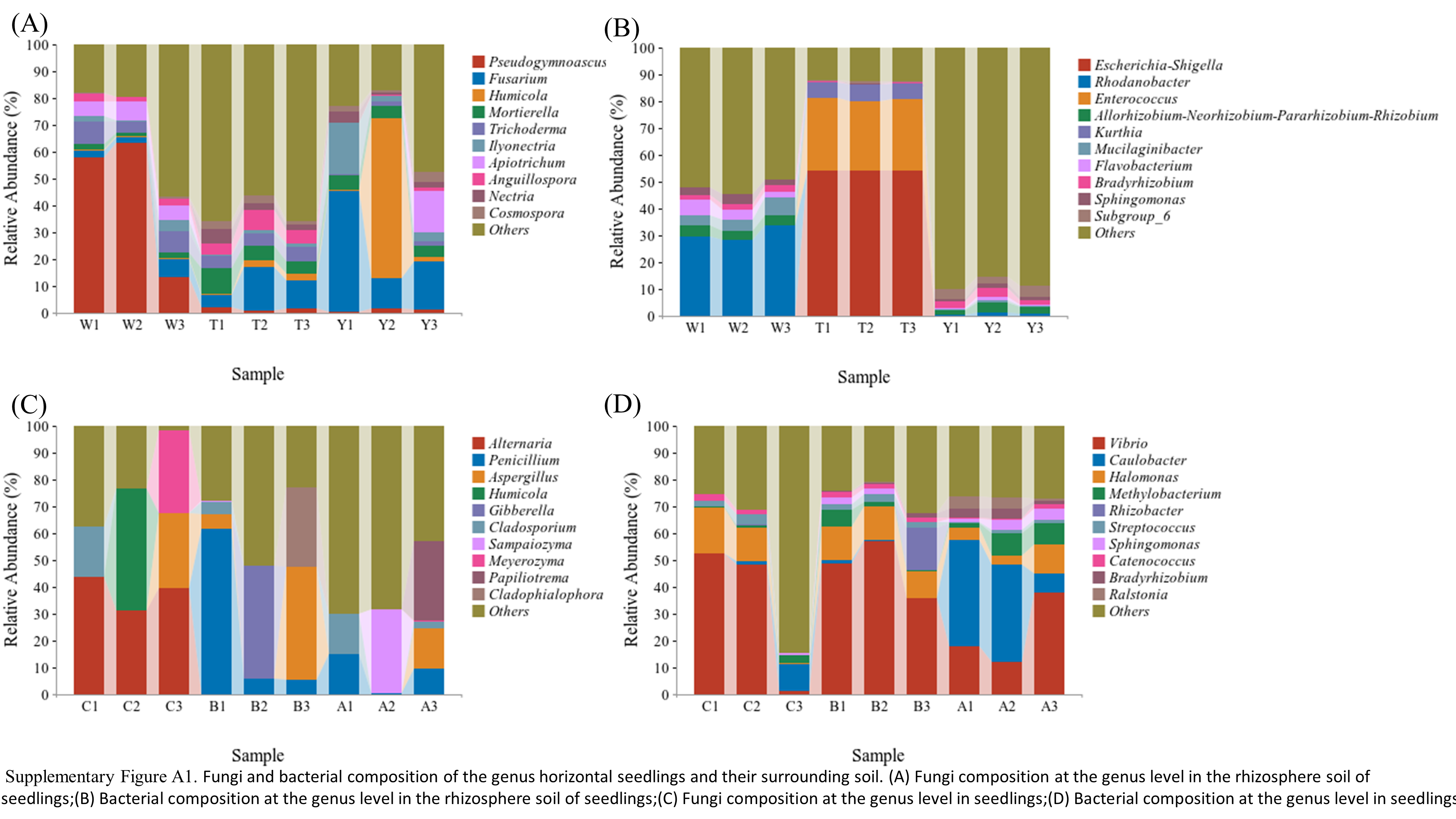

Supplement: Supplementary file 1 [file DataSheet_1.zip › Supplementary Figure A1.tif]

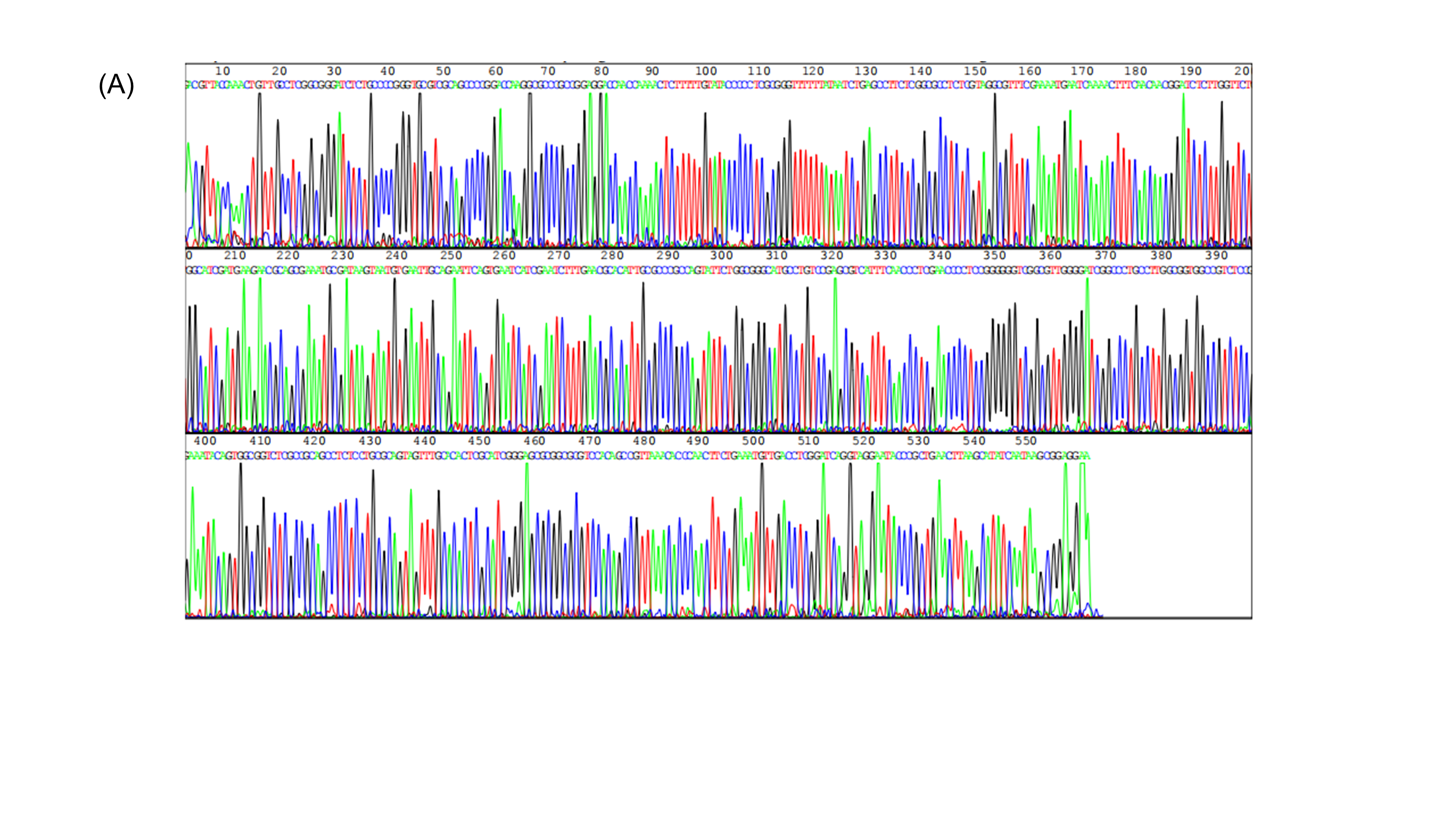

Supplement: Supplementary file 1 [file DataSheet_1.zip › Supplementary Figures A2A.tif]

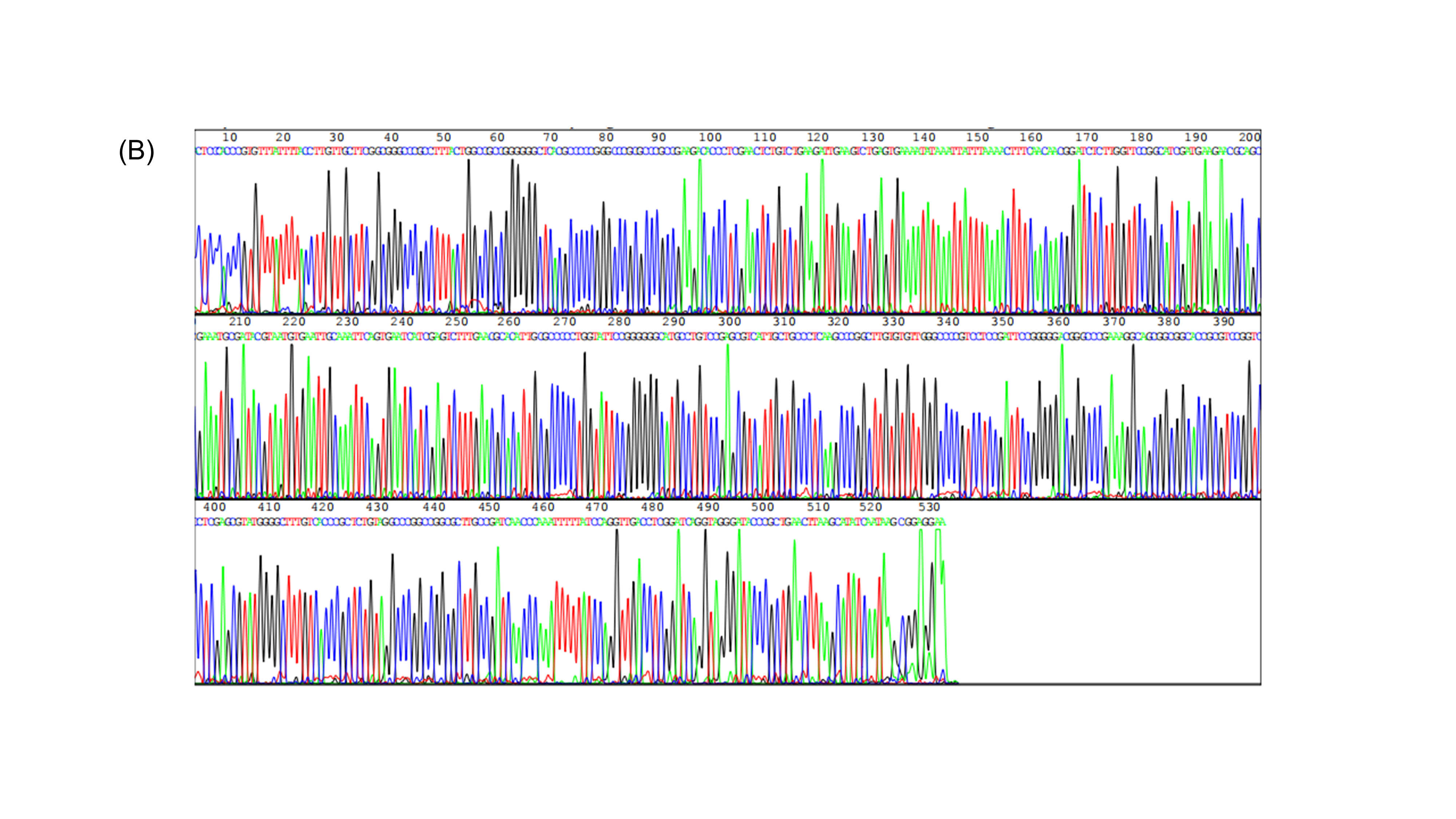

Supplement: Supplementary file 1 [file DataSheet_1.zip › Supplementary Figures A2B.tif]

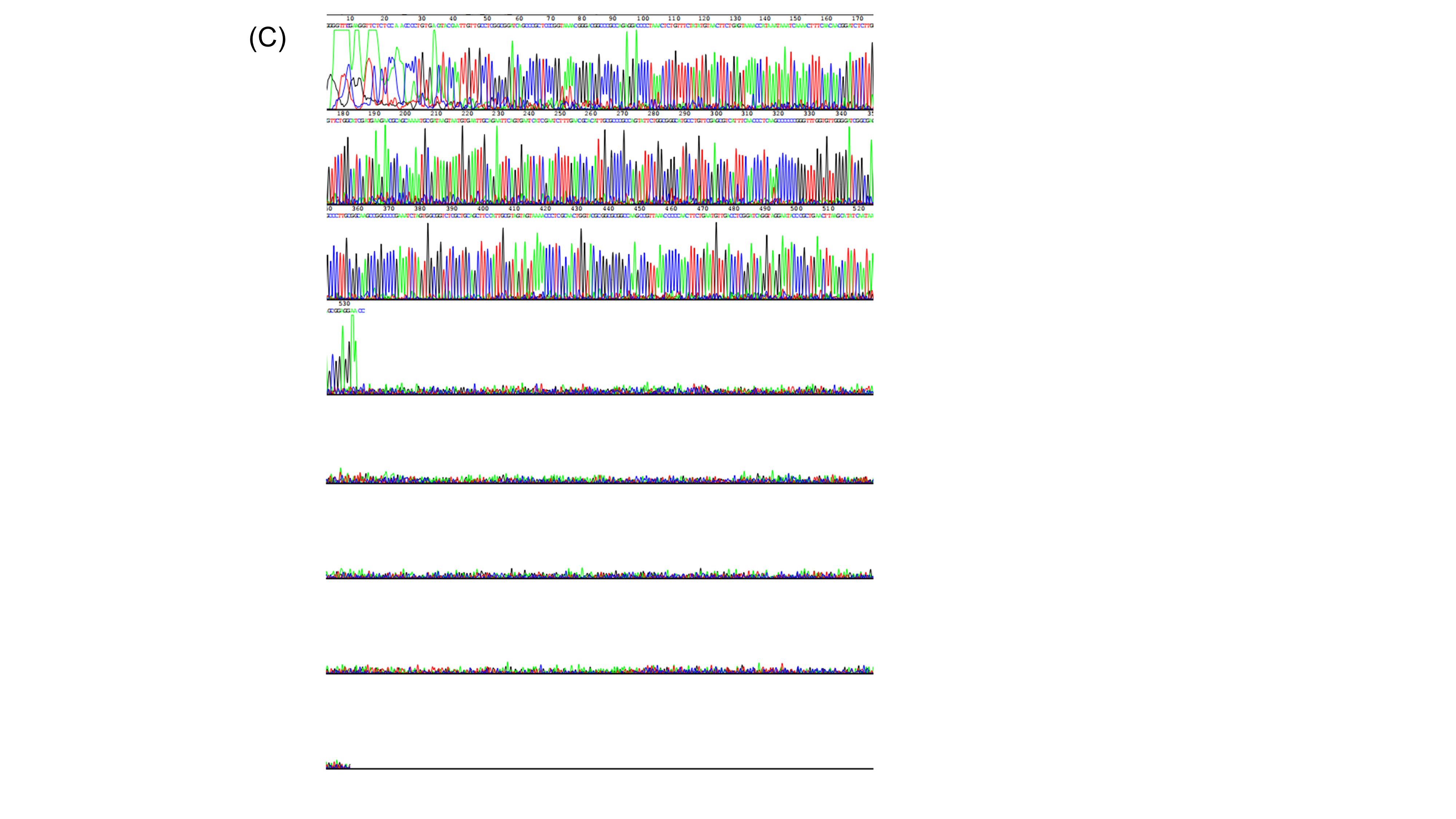

Supplement: Supplementary file 1 [file DataSheet_1.zip › Supplementary Figures A2C.tif]

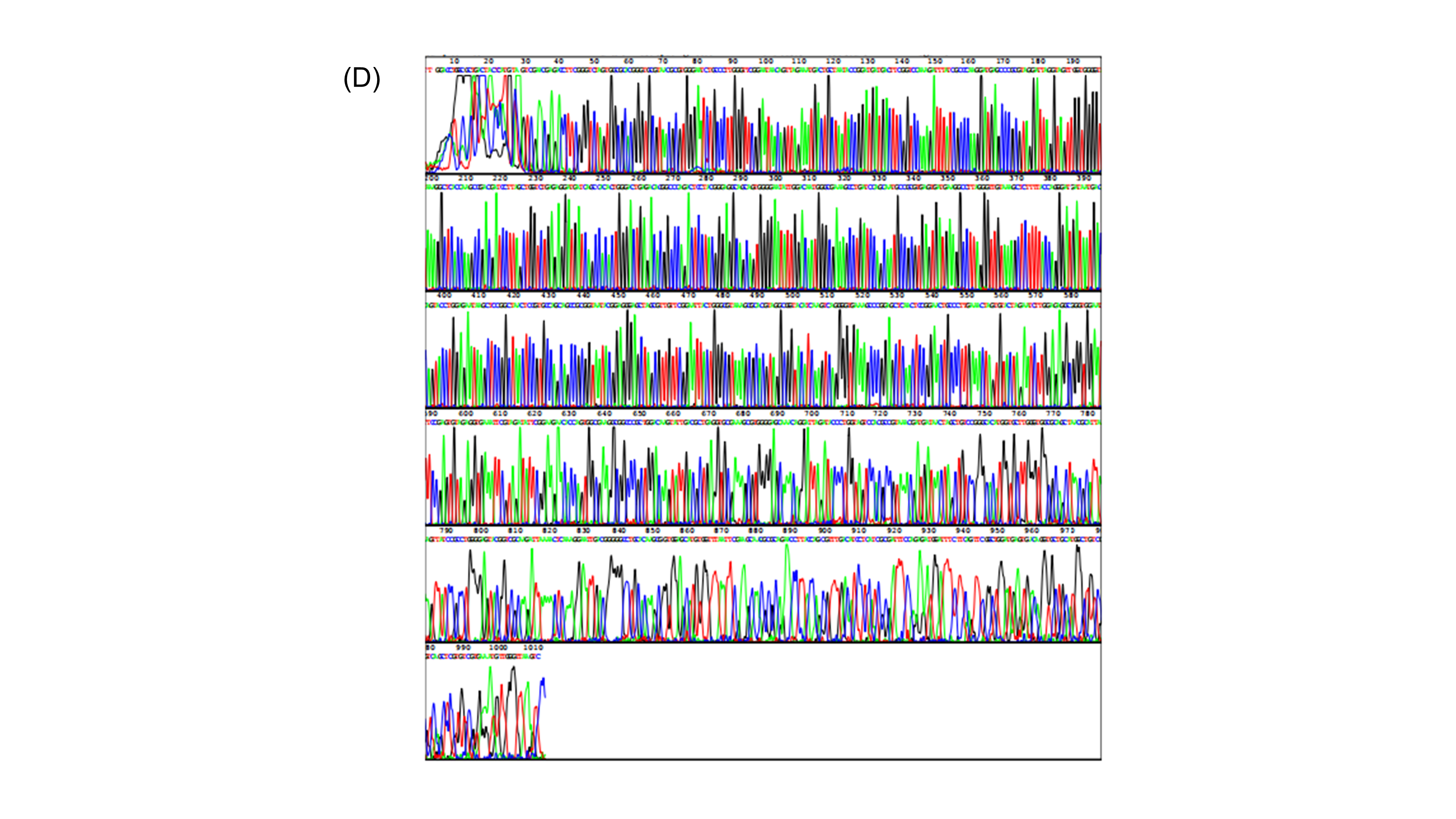

Supplement: Supplementary file 1 [file DataSheet_1.zip › Supplementary Figures A2D.tif]

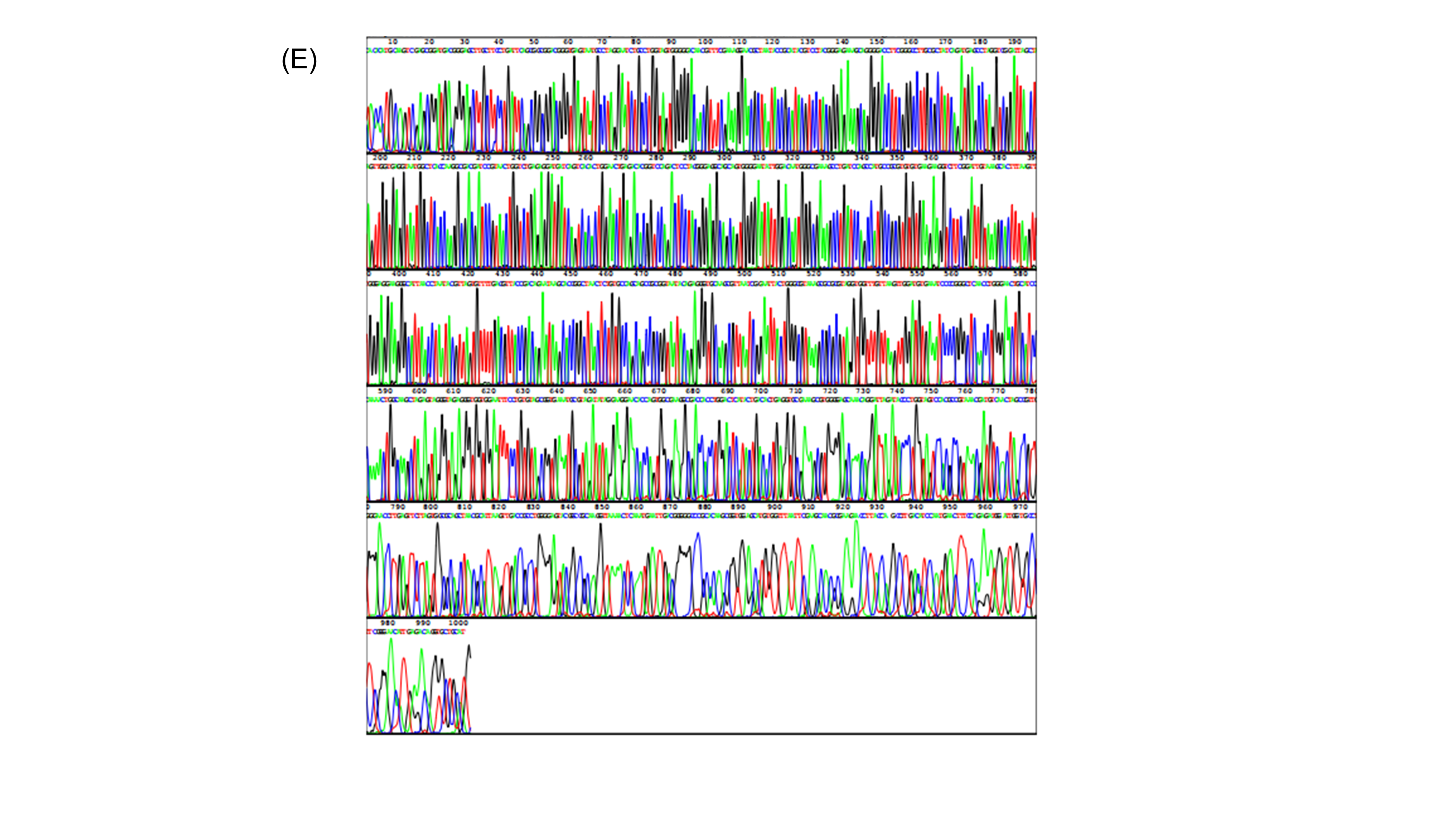

Supplement: Supplementary file 1 [file DataSheet_1.zip › Supplementary Figures A2E.tif]

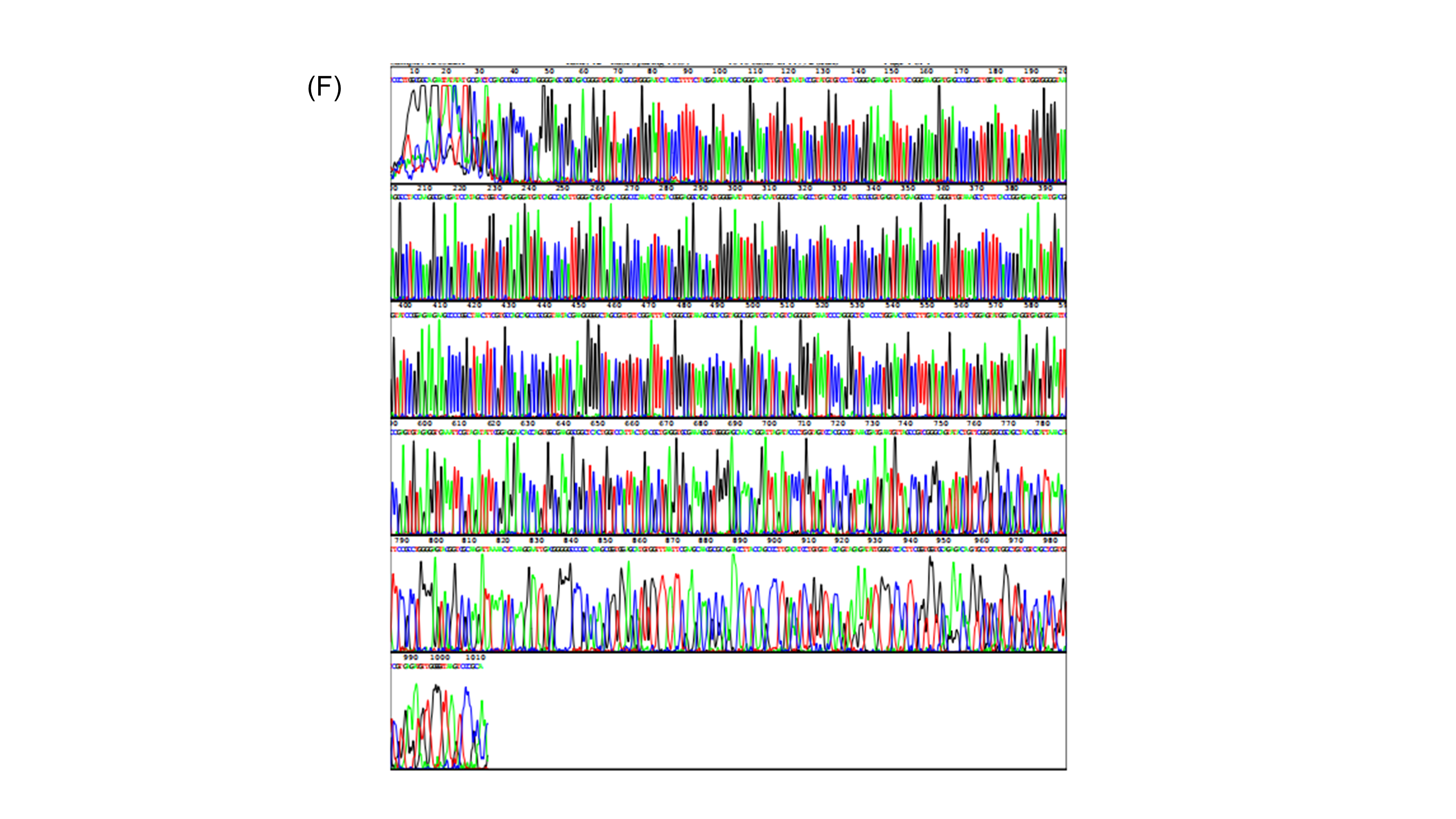

Supplement: Supplementary file 1 [file DataSheet_1.zip › Supplementary Figures A2F.tif]

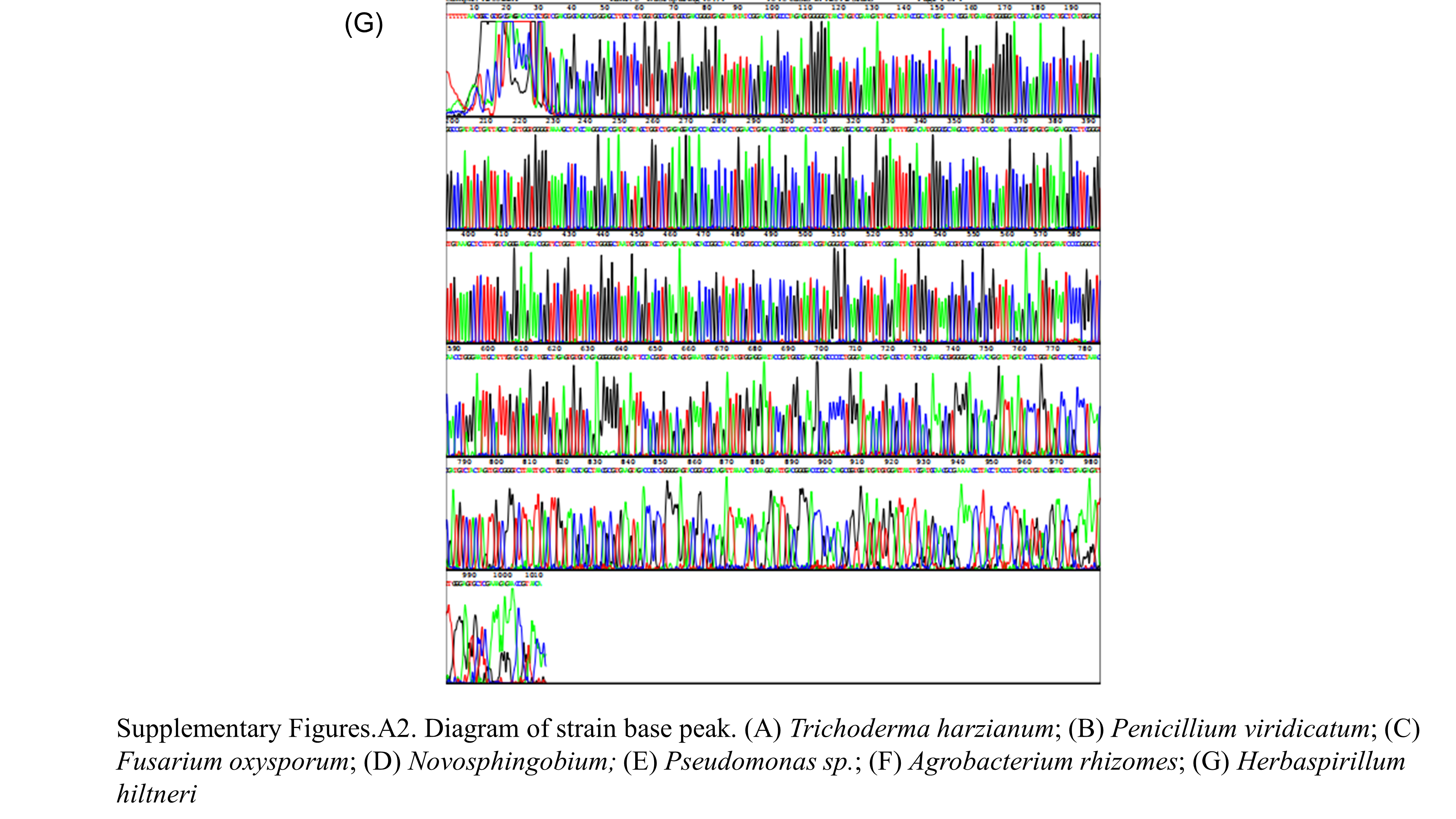

Supplement: Supplementary file 1 [file DataSheet_1.zip › Supplementary Figures A2G.tif]

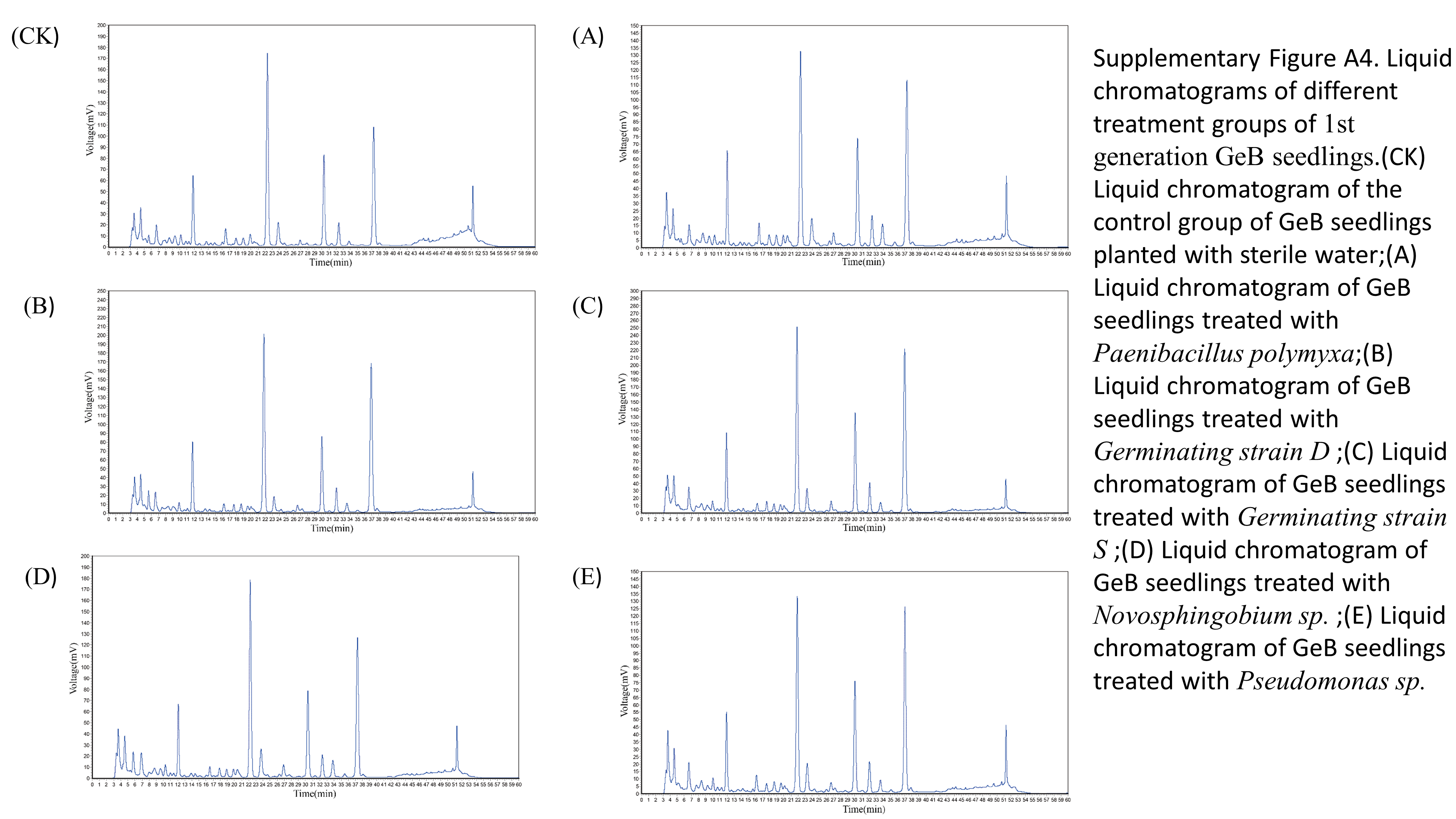

Supplement: Supplementary file 1 [file DataSheet_1.zip › Supplementary Figure A4.tif]

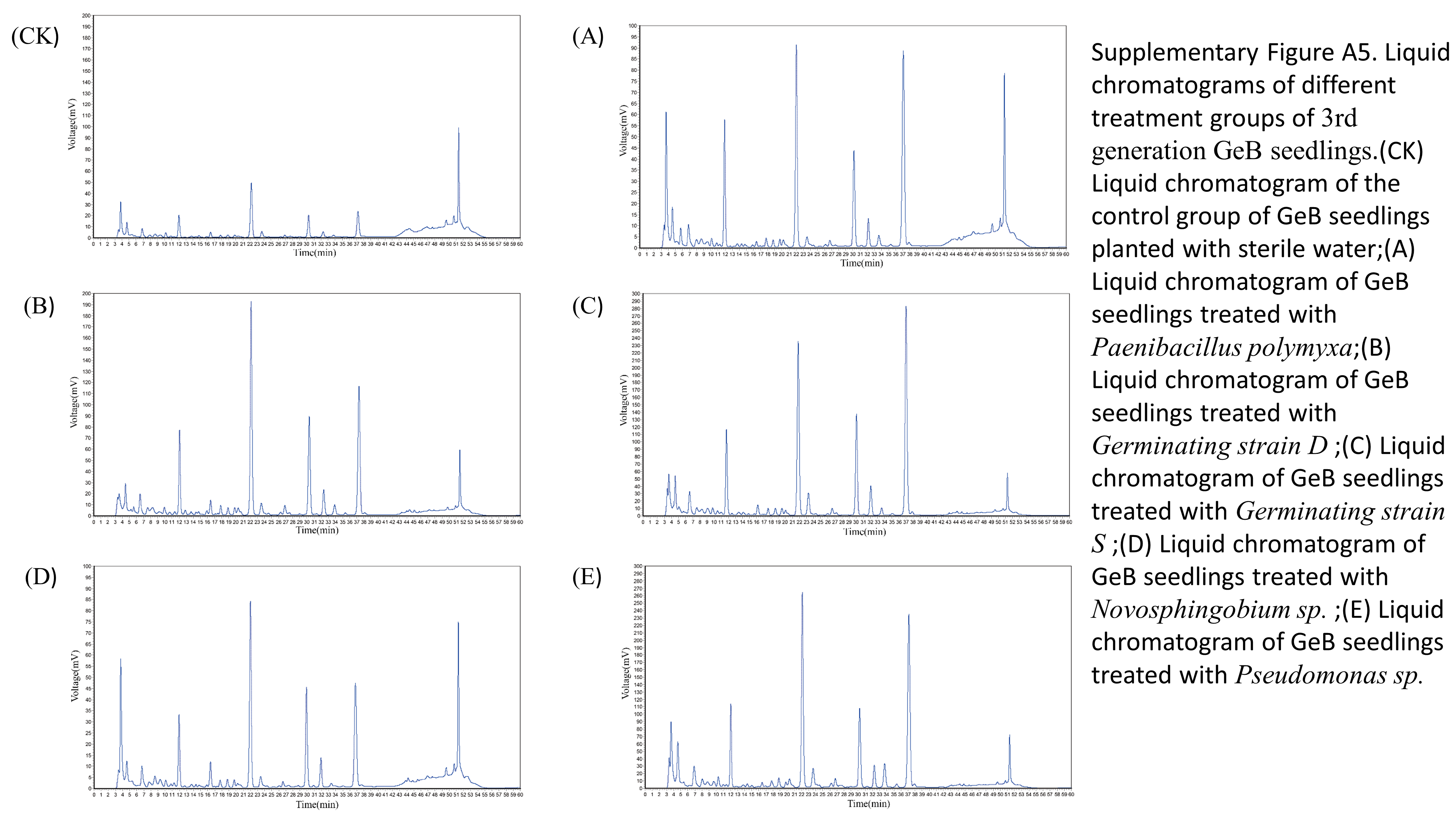

Supplement: Supplementary file 1 [file DataSheet_1.zip › Supplementary Figure A5.tif]

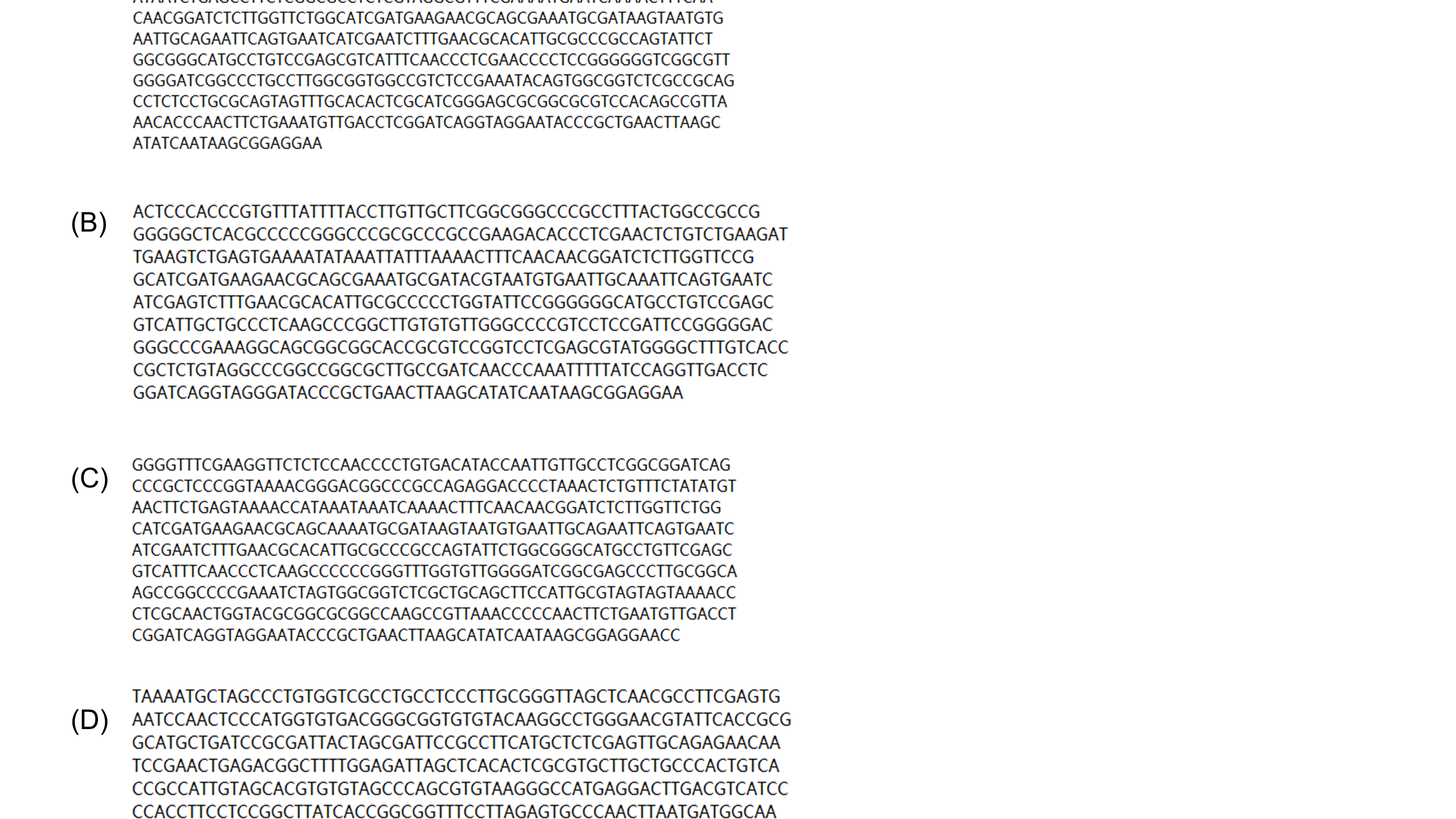

Supplement: Supplementary file 1 [file DataSheet_1.zip › Supplementary Figures A3A-D.tif]

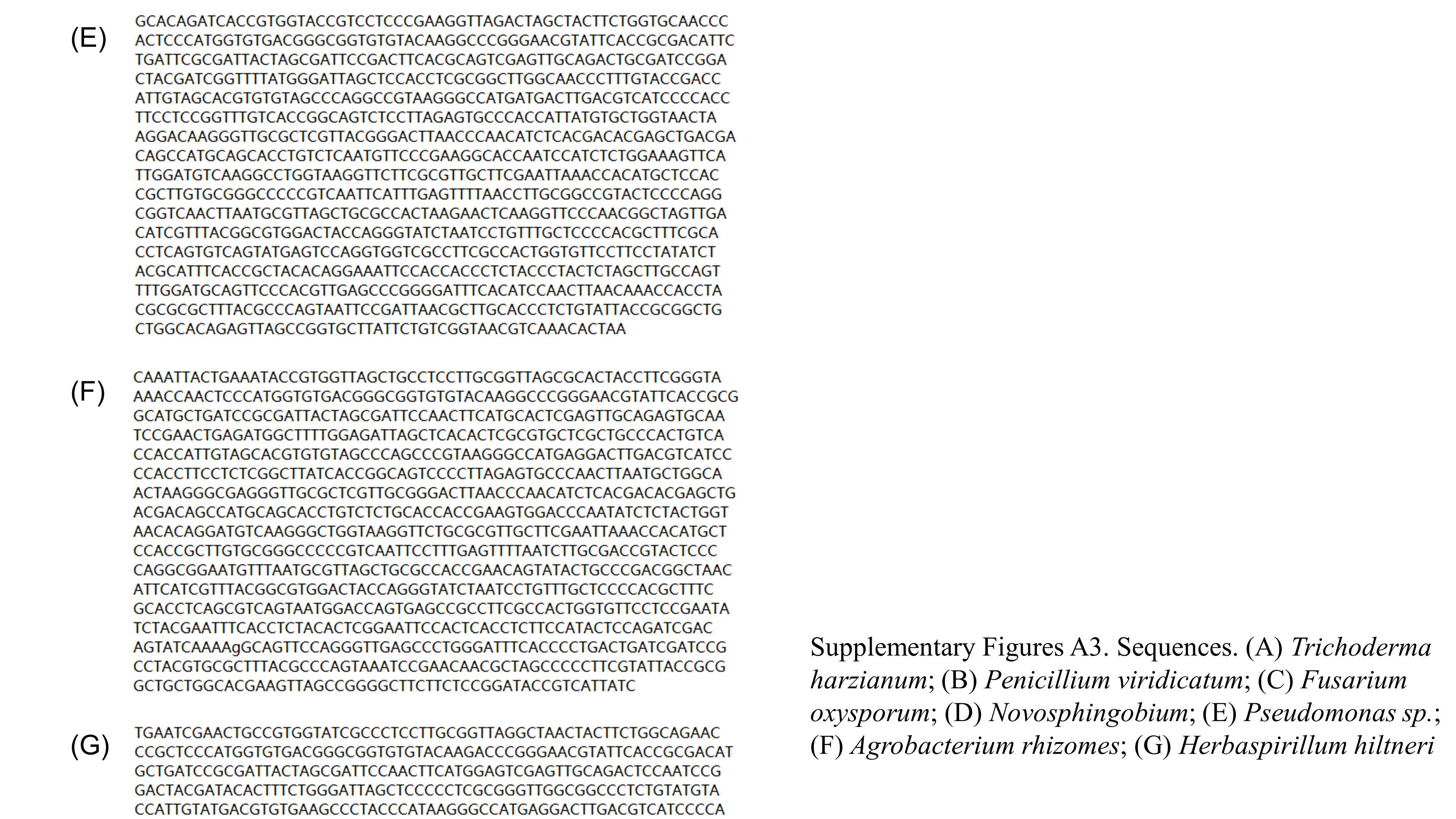

Supplement: Supplementary file 1 [file DataSheet_1.zip › Supplementary Figures A3E-G.tif]
